# Optimally sequencing semantic search predicts creativity

**DOI:** 10.1371/journal.pone.0352328

**Published:** 2026-06-24

**Authors:** Olivier Toubia, Jonah Berger

**Affiliations:** 1 Columbia University, New York City, New York, United States of America; 2 University of Pennsylvania, Philadelphia, Philadelphia, United States of America; Museo Storico della Fisica e Centro Studi e Ricerche Enrico Fermi, ITALY

## Abstract

Creativity is a fundamental human activity which drives progress and innovation. Extant research has documented the link between creative performance and the efficient navigation of semantic space during the creative process, but we argue that an important aspect of efficiency has been overlooked: the optimal sequence of concepts retrieved. We develop a simple cognitive test, the Shortest Semantic Path Task, and an associated automatic measure (circuitousness), which capture this insight. Five initial validation studies demonstrate that this novel measure predicts creativity above and beyond existing measures, providing a more complete picture of creative performance. The psychometric properties of the specific instruments tested also reveal opportunities to develop more robust and consistent instruments to measure optimal sequencing in semantic search.

## Introduction

Creativity is a quintessential human activity, and the source of scientific, artistic, cultural and industrial progress [1]. Accordingly, a large literature has developed to understand, quantify and nurture creative performance [2].

The efficient retrieval of relevant concepts plays a key role in the creative process [3]. Indeed, when solving complex creative problems, people tend to sequentially retrieve multiple relevant concepts that are connected to each other. When generating ideas for cell phone apps to help users be healthier, for example, people need to retrieve relevant concepts which, appropriately combined, form a novel and useful idea. One may activate concepts of pictures, loyalty points, and healthy smoothies, for example, and combine them to suggest users take pictures of completing healthy tasks in exchange for points they can redeem at participating vendors for free healthy smoothies. The concepts retrieved during semantic search serve as input for the generative phase of the creative process in which these concepts are combined in meaningful ways [4]. People who retrieve more relevant concepts with limited cognitive effort are able to explore more potential ideas with the same cognitive resources, and should therefore perform better at creative tasks.

Starting with [5]’s groundbreaking work, creativity and the ability to retrieve relevant concepts with limited cognitive effort have often been associated with flat associative hierarchies that allow individuals to move *effortlessly* around semantic spaces. More recent research has shown that creative individuals have memory structures that allow them to search through semantic space more fluently and to make more distant associations (e.g., [3,6]). This line of research has led to the development of several tasks that measure divergent thinking or people’s ability to “generate a broad range of solutions or ideas to a given stimulus” [7, Page 307]. Tasks like the letter fluency task [8] or the Unusual Uses test [9], for example, ask participants to retrieve distinct concepts that are all related to one focal concept (e.g., alternate uses for a wire hanger). This requires an ability to retrieve and evaluate many mutually distant yet connected concepts. Tasks like the Forward Flow Task [10] or the Divergent Association Task [11] measure this ability more directly. Highly creative individuals perform better at these tasks because they have a semantic memory structure that facilitates the retrieval of more distant concepts [5,10,12,13].

**Fig 1:**
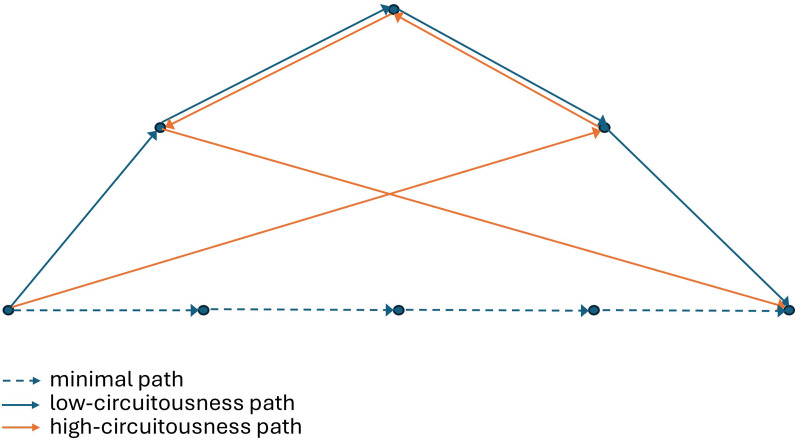
Conceptual Illustration of semantic paths.

A useful analogy for thinking about semantic search in the creative process is that of animals foraging for food. Indeed, [14] argue and demonstrate that semantic search is similar to search in physical space, involving a dynamic process comparable to that of animals foraging among patches of food in their environment. Further tests of this theory were provided, among others, [15, 16]. This analogy makes it is clear how moving effortlessly through semantic space allows visiting disparate areas (patches of information) without too much effort, thus improving the output of semantic search and facilitating exploration [16]. Fig 1 illustrates this insight. A low cost of effort makes it easier to take longer paths (e.g., blue path), allowing exploration of the space.

Beyond effort being less costly, however, the return on effort (i.e., spending it parsimoniously and efficiently) should also facilitate creativity. Indeed, research has shown that creative cognition involves not only “default” semantic network activity (which facilitates the retrieval of potentially relevant information from long-term memory), but also executive control to constrain cognition and meet specific task goals [17,18]. This suggests that while semantic structure determines the availability of associations, executive processes determine the efficiency with which those associations are navigated. In particular, [3] argue that performance on creative thinking tasks requires extracting information from long-term memory while *avoiding repetitions*.

While this insight has not received much attention in the literature, we argue it may also be generalized to the concept of *optimal sequencing in semantic search*. While [3] focus on repeating specific words, we argue that optimal semantic search should in general avoid repeating “semantic territory.” Just as verbal fluency requires executive control to avoid redundant outputs (repetitions), optimal semantic search requires executive control to avoid “navigational redundancy,” i.e., search paths that revisit semantic areas already traversed. Relying again on the foraging analogy, while a low cost of effort may translate into being able to move between patches more easily, spending effort parsimoniously and avoiding navigational redundancy require the optimal *sequencing* of the information patches visited during semantic search. This relates to the problem of optimal movement between patches, a critical but understudied component of foraging theory [19].

For example, the orange path in Fig 1 visits the same points as the blue path, i.e., it does not explore the space more or less. However, because the points in the orange path as sequenced suboptimally, this path requires more effort (longer path) to explore the same points in the space. That is, efficiency in semantic search should come not only from a lower cost of effort (i.e., flatter hierarchies that allow taking bigger leaps with less cognitive effort), but also from spending effort more efficiently by optimally sequencing the retrieval of concepts and not wasting “semantic effort” unnecessarily.

Optimal sequencing during semantic search should in turn be relevant and useful in any stage of the creative process that relies on associative thinking [3]. It is defined conceptually as the ratio of the semantic distance travelled during semantic search, i.e., the distance between consecutive concepts retrieved, to the minimum semantic distance needed to connect these concepts if they were optimally sequenced (i.e., in a way that minimizes total distance travelled).

While optimal sequencing in semantic search makes conceptual sense, we are not aware that of any literature that has examined it. It is not captured by extant tests of divergent thinking, or by traditional tests of convergent thinking (generally defined as the ability to discern solutions “with the objective of arriving at a single, correct solution,” [7]).

Accordingly, we developed a task that measures, in a quantitative and systematic manner, the optimal sequencing of concepts while performing semantic search. Exploratory studies suggest that this task improves the quantification and prediction of creativity while being distinct from traditional measures of creative abilities. For example, if optimal sequencing were simply a reflection of flatter associative hierarchies, it should be highly correlated (e.g., r > 0.70) with Forward Flow [10] or the Divergent Association Task [11]. Analyses, however, indicate low correlations (r < 0.1), suggesting it is independent. While these initial results are promising, we note that the independence between associative flatness and optimal sequencing remains a preliminary finding that requires further corroboration and replication in independent studies.

### The Shortest Semantic Path Task (SSPT)

**Fig 2:**
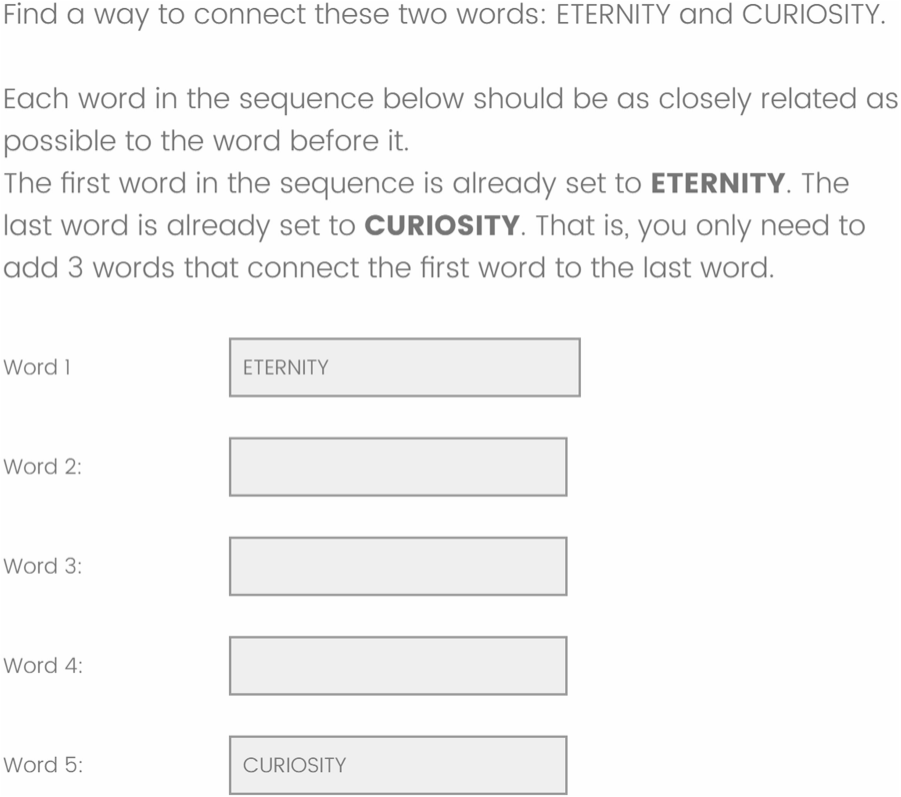
The shortest semantic path task.

To measure optimal sequencing of semantic paths, we developed a simple, scalable task that asks participants to find a short path connecting two random words. The SSTP asks participants to connect two random seed words (first and last words in the sequence), i.e., find a short semantic path that connects two relatively distant concepts. Fig 2 shows an example, where the participant is asked to connect the words “Eternity” and “Curiosity,” and where the participant constructs the following path: “eternity” → “life” → “human” → “mind” → “curiosity.”

In addition to measuring the optimality of sequential semantic search, SSPT offers several additional benefits. First, it lends itself to automatic scoring. As we discuss next, it is easy to use standard word embeddings to develop relevant measures that describe the set of words retrieved by a participant. Second, it is easily scalable, as any words may be used as seeds. This means that unlike other tasks like the Remote Associates Tasks, that have final question banks, researchers can create practically infinite versions of this task. Third, instead of having a “correct” answer that participants either get correct or not (binary outcome), this task leads to a wide range of outcomes that can be scored continuously. This provides finer, continuous measures of performance. It also means that there is no concern of a person, or a large language model, having memorized the correct answer to a particular version of this task. Indeed, there is a growing literature trying to quantity the creative abilities of large language models (e.g., [20–23]). Asking a large language model to complete tasks like the Remote Associates Task would be problematic due to leakage: these tasks may be in the training data of these models, meaning that the correct answer may be directly accessible to them. In contrast, researchers could administer the SSPT to a LLM using random sets of words. Even if future versions of LLMs become “aware” of this task, it is highly unlikely they would have been trained on the exact set of random words selected by the researcher.

### Circuitousness

We capture the efficiency of one’s semantic path when solving the SSPT using word embeddings. While various tools can represent words in a latent semantic space, given its prevalence in the literature, we rely on word2vec [24]. Throughout the paper we use the classic “word2vec-google-news-300” model, trained on about 100 billion words from the Google News dataset, containing vector for 3 million unique words and phrases, and with a vector dimensionality of 300, accessible via the Gensim library in Python. We do not use vector normalization. A participant’s answers can be captured by five vectors in the latent semantic space: {*w*_*1*_, *w*_*2*_, *w*_*3*_, *w*_*4*_, *w*_*5*_}, where each vector represents the corresponding word in that space (*w*_*1*_ and *w*_*5*_ are set by the researcher, and the participant’s input consists of *w*_*2*_, *w*_*3*_, *w*_*4*_*).* If any of the word in the sequence is not recognized by Word2vec, circuitousness is considered missing for that task. This happened in 3.75% of the observations in Study 1, 5.78% in Study 2, 7.54% in Study 3, 4.14% in Study 4a, and 4.11% in Study 4b. This was typically due to misspelling, or rare words or bi-grams (e.g., “saber-tooth”).

**Fig 3:**
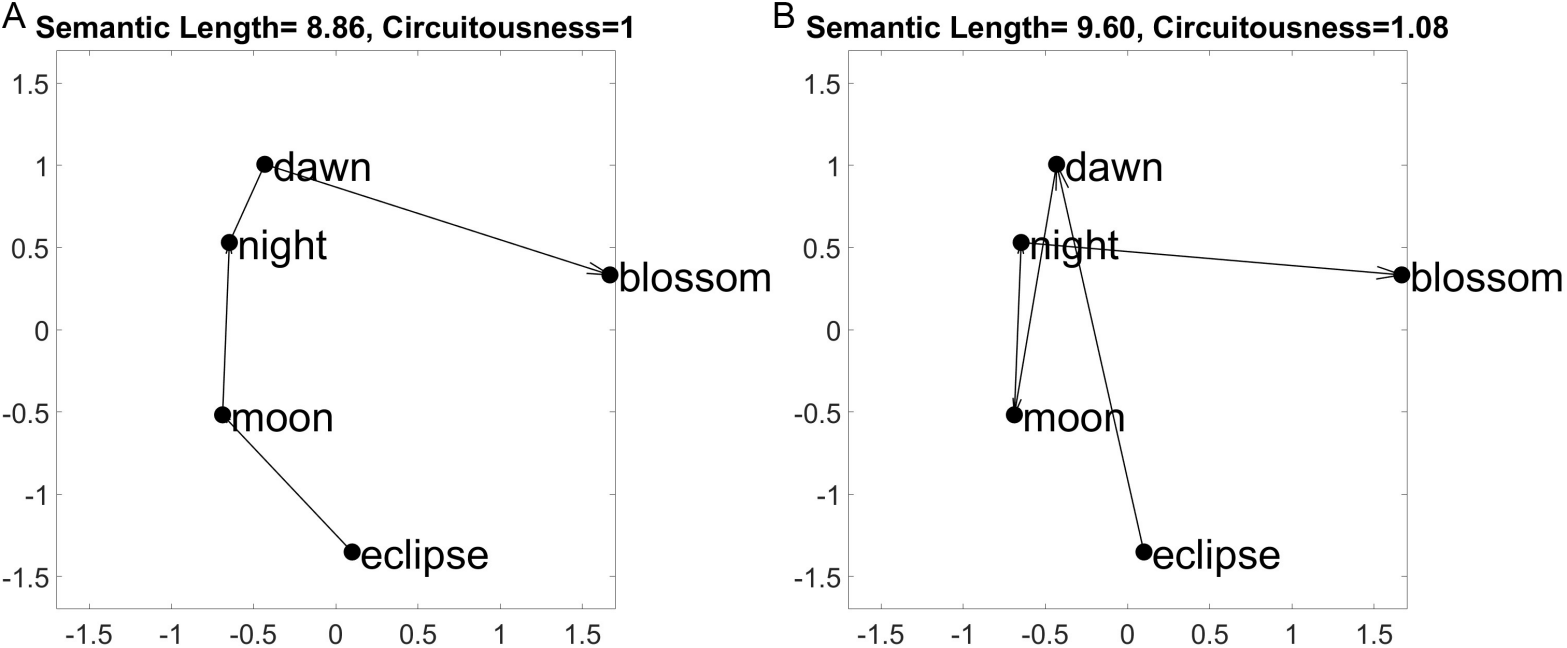
Semantic path illustration with actual words.

Suppose the first word is “eclipse,” for example, the last word is “blossom,” and the intermediate words are “moon,” “night” and “dawn.” Fig 3 illustrates the semantic paths eclipse → moon → night → dawn → blossom vs. eclipse → dawn → moon → night → blossom. Following [25], we visualize the paths by applying Principal Component Analysis on the set of embedding vectors corresponding to the five points, and plotting the first two components. This indicates that the points are ordered optimally in the first path (left panel), which is the shortest possible path that starts at “eclipse,” ends at “blossom,” and visits “moon,” “night” and “dawn.” The second path (right panel), in contrast, is unnecessarily long and inefficient, spending more semantic distance to cover the same set of points.

Formally, we use *circuitousness* to measure the optimality of the sequence of concepts retrieved by a participant when solving the SSPT. Following [26], we define circuitousness by the extent to which the semantic path retrieved by the participant differs from the shortest path that starts at *w*_*1*_, ends at *w*_*5*_, and visits *w*_*2*_, *w*_*3*_ and *w*_*4*_ in between (in any order). The shortest semantic path is found by solving a version of the Traveling Salesperson Problem [27]. Given the small dimensionality of our problem, we solve the Traveling Salesperson Problem by enumeration. Mathematically, we define: circuitousness = (length of semantic path)/(length of shortest semantic path). In Fig 3, the path on the left has a circuitousness of 1 because it is optimal, and the path on the right has a circuitousness of 1.08 > 1. Note that [26] adopt a log-scale and define: circuitousness = log((length of semantic path)/(length of shortest semantic path)). We do not take the log scale here to improve the comparison with other metrics such as Forward Flow and Divergent Association Task. Similar results are obtained if we log-transform circuitousness.

Note that it might be tempting to simply consider the length of the actual path submitted by the participant. However, our theoretical discussion above suggests that the semantic paths retrieved by creative individuals may *not* necessarily be shorter than the paths retrieved by less creative ones. Indeed, high creative individuals are better able to retrieve distant concepts [3,5,6] and hence may tend to retrieve concepts that are further apart. In addition, creative individuals tend to underestimate the semantic distance between concepts [28] which could lead them to form paths that they believe are short but that are not as short as they think. Moreover, as discussed above, longer semantic paths may be a sign of an ability to explore the semantic space [16], which may promote creative achievements. Indeed, [29] find that more creative individuals tend to travel further in the semantic network when engaging in free association. In sum, while we do not expect more creative individuals to retrieve shorter semantic paths, we do expect them to retrieve less circuitous (i.e., more optimally sequenced) ones. We empirically contrast circuitousness with path length in the next section.

We also note that submitted semantic path does not need to reflect the participant’s exact search process, i.e., participants have the ability to reorder the words and submit a path that does not reflect the order in which the words were retrieved. However, empirical analyses indicate that participants entered words in their natural order (i.e., the second, third and fourth words were entered in that sequence) in most cases (i.e., 88.10% of tasks in Study 4a and 86.36% in Study 4b – the two studies where order was recorded).

Further, while one might wonder whether the SSPT could be described as a version of convergent thinking, it is conceptually and empirically distinct. Definitions of convergent thinking differ across authors, but [11]’s operationalization of convergent thinking tasks as those that “measure the ability to assess several stimuli and arrive at the most appropriate response, such as the optimal solution to a problem” (Page 1), could be viewed as covering the SSPT. Our task specifically focuses on *sequential* semantic searches, however, and the *optimality* with which participants sequence concepts in them. Further, results indicate our measure adds to the ability to predict creative performance, even after controlling for a typical measure of convergent thinking (i.e., performance on the Remote Associates Test). An overarching question is whether the definition of convergent thinking is perhaps too broad (measuring the ability to solve creative tasks by the ability to find optimal solutions to problems) and whether more granular theories, tasks, and measures are needed to provide a rich picture of someone’s creative performance.

We tested the SSPT and circuitousness measure in five initial pre-registered validation studies (As Predicted #169975, #168162, #177345, #272,931, #278,393). Given the novelty of the task and associated measure, we focus on predictive validity (i.e., whether circuitousness is able to predict creative performance on an idea generation task), discriminant validity (i.e., whether it correlates strongly with standard measures and whether the link with creativity persists after controlling for them), and other psychometric properties (e.g., test-retest reliability). In Studies 1-4a, participants completed a target idea generation task proposing a “new idea for a smartphone app priced at $0.99 that would help its users keep a healthier lifestyle.” Creativity ratings on this task (obtained from a different set of participants, with approximately 20 creativity ratings per idea) served as the main creativity measure. Study 1 (N = 320) explored the intercorrelations between the SSPT, a letter fluency task [8], Guilford’s Unusual Uses test [9], and the Remote Associates Test [5,30] as well as how they correlate with creativity. Participants who spent significant time on other windows were removed, but given the use of online participants in Study 1, one could still be concerned about reliance on Generative AI tools. Consequently, Study 2 (N = 128) used a more supervised laboratory setting. Study 3 (N = 122) was similar to Study 2 but included additional benchmark tasks: the Divergent Association Task [11] and the Forward Flow task [10]. Study 4a (online, N = 442) included a crystallized intelligence test [31] and 10 items in the SSPT to explore split-half reliability (a form of internal consistency). Study 4b (online, N = 365) explored test-retest reliability by inviting the participants who completed Study 4a to complete the same 10 items of the SSPT in the same order, 4 weeks later. All data are available at https://researchbox.org/4627 (Studies 1–3) and https://researchbox.org/6472 (Studies 4a-b). Analysis code is available from the corresponding author upon request.

## Materials and methods

All studies were implemented on Qualtrics (Qualtrics surveys will be shared with researchers interested in using our instruments). Studies 1, 4a and 4b were run online on Prolific. Studies 2 and 3 were run in person in Columbia University Behavioral Research Lab. Overall, the lab’s participant pool primarily consists of students (approximately 45% undergraduate, 45% graduate, and 10% non-students), with approximately 2/3 identifying as female. All studies were conducted in compliance with the Institutional Review Boards of Columbia University’s Human Research Protection Office under Protocol IRB-AAAV1452 (approved 04/02/2024). Informed consent was obtained online before the start of each study. The recruitment period across the five studies started on 04/09/2024 and ended on 03/18/2026. Screenshots from each task are included in the SI.

In each study, participants first completed all tasks except the idea generation task in a randomized order (i.e., all permutations were equally likely), followed by the idea generation task (in Studies 1-4a). Below is a description of each task.

### Shortest Semantic Path Task

After one practice round (in which the seed words were “eclipse” and “blossom” and the proposed intermediate words were “moon”, “night”, and “dawn”), each participant in Studies 1–3 completed five rounds with the following pairs of seed words: {eternity, curiosity}, {elephant, galaxy}, {perseverance, eloquence}, {euphoria, tulip}, {tangerine, penguin}, where rounds were presented in a random order (all permutations equally likely). Participants in Studies 4a completed 10 rounds: the first five (also presented in random order) used the following pairs of seed words: {mountain, clock}, {midnight, echo}, {river, silver}, {table, ocean}, {gravity, velvet}; the last five (again presented in random order) used the same set of seed words as in Studies 1–3. Participants in Study 4b completed the same 10 rounds as in Study 4a in the same order (i.e., the order of rounds was identical across the two studies for each participant). In each task, participants were able to delete and correct their words before submitting their answers. Answers containing duplicate entries (i.e., not all five words in the sequence were distinct) were discarded (this happened in 0.38% of observations in Study 1, 0.16% in Study 2, 0.00% in Study 3, 0.07% in Study 4a, 0.33% in Study 4b). In each study, circuitousness was standardized separately for each task (defined by a pair of seed words) across all valid entries for that task in that study, and averaged across rounds to form the final measure.

### Letter fluency task [8]

Participants were given 2 minutes to list as many words that begin with the letter F as possible, without consulting a dictionary or any other source. Performance at this task has been shown to correlate both with verbal ability and control ability [32]. Performance was measured by the number of unique valid words submitted.

### Unusual Uses Test [9]

Participants were asked, without consulting a dictionary or any other source, to list as many different unusual uses for a wire coat hanger as possible, in 2 minutes. Performance was measured as the creativity of the set of uses submitted by the participant. Results are robust to controlling for the number of uses (see SI). We obtained creativity ratings using standard paradigm in the idea generation literature (e.g., [33,34]). We recruited participants from Prolific, asking each to rate 20 randomly selected responses, where each response contains all uses suggested by one participant in the main study. The instructions were: “This participant was asked to list as many different unusual uses for a wire coat hanger as possible. How creative is this response overall?”. Creativity was rated on a five-point scale from “very uncreative” to “very creative.” Participants were not trained beyond these instructions, did not receive examples of creative vs. non-creative responses, and there was no calibration process among raters. This paradigm focuses on collecting a larger number of creativity ratings for each idea from a larger set of raters, rather than a smaller set of ratings from a smaller set of raters. We targeted 20 evaluations per response, and collected an average of 20.60 in Study 1 (standard deviation = 4.38); 20.10 in Study 2 (standard deviation = 4.16); and 20.63 in Study 3 (standard deviation = 4.18). The creativity of the set of uses submitted by a participant is measured as the average of creativity rating across judges. Given each submission (target) was rated by a different set of judges, we assess inter-judge reliability by computing an IntraClass Correlation (ICC) using a one-way random effects model [35], and find ICC(1,k)=0.91 in Study 1, 0.92 in Study 2, and 0.89 in Study 3.

### Remote Associates Test [5,30]

Following one practice round, each participant completed eight rounds, with 15 seconds to complete each. Stimuli were taken from [36]’s set of problems and we picked problems with various levels of difficulty. The triplets of remote associate items and the associated solutions were: cream/skate/water: ice; worm/shelf/end: book; palm/shoe/house: tree; tank/hill/secret: top; tail/water/flood: gate; iron/shovel/engine: steam; horse/human/drag: race; hungry/order/belt: money. Performance on this task was measured by the number of correct answers.

### Divergent Association Task [11]

Participants were asked, “without consulting a dictionary or any other source, to write 10 words that are as different from each other as possible, in all meanings and uses of the words.” Following [11], the first seven valid (i.e., recognized by Word2vec) words provided by the participant were retained. Performance on the task was measured as the average pairwise cosine distance between each 21 pair of words using Word2vec embedding vectors.

### Forward Flow [10]

Starting with an initial seed word (selected randomly among {candle, snow, toaster, paper, table, bear} as in [10]), participants were instructed to “write down the next word that follows in your mind from the previous word.” Participants were instructed to write 20 words (including the seed word). We compute the cosine distance between each pair of words, based on their Word2vec embedding vectors and performance is measured as the average pairwise cosine distance between words. This measure is limited to words recognized by Word2vec. In our data there was no instance of a word not recognized by Word2vec in this task.

### Crystallized Intelligence Test [31]

To further assess whether differences in circuitousness simply reflect more general differences in verbal ability, Study 4a included a crystallized intelligence test, containing 20 multiple-choice questions. Ten asked participants to identify a synonym to a target word from a candidate set, and ten asked them to identify antonyms. The detailed list of words is included in the SI. The average number of correct responses was 12.52 (std = 4.91).

These tasks were followed by an idea generation task.

### Idea Generation

We used the following prompt: “We are interested in new ideas for smartphone apps that will help their users keep a healthier lifestyle. In the space provided below, please enter one new idea for a smartphone app priced at $0.99 that would help its users keep a healthier lifestyle. Please be as specific as possible and describe the main features of the app.” Participants were able to submit their idea only if it contained a minimum of 200 characters. Ideas were reviewed by the first author to remove obvious “junk” ideas that contained offensive language (e.g., “Honestly I hate you for using prolific as a means to gather creative ideas”) or that were clearly off topic (e.g., an app for education rather for health) and participants with junk ideas were removed.

### Creativity Scores

Creativity scores were obtained from a different set of participants, using a standard paradigm in the idea generation literature (e.g., [33,34]). We recruited participants from Prolific, asking each to rate 20 randomly selected ideas. Participants were informed that “We will show you ideas for smartphone apps priced at $0.99 that would help their users keep a healthier lifestyle. You will be shown 20 ideas, one after the other. Your task is to judge how creative each idea is,” and for each idea were asked: “How creative is this app idea?”. Creativity was rated on a five-point scale from “very uncreative” to “very creative.” Participants were not trained beyond these instructions, they did not receive examples of creative vs. non-creative responses, and there was no calibration process among raters. We targeted 20 ratings per idea, and collected an average of 20.94 in Study 1 (standard deviation = 4.33); 19.61 in Study 2 (standard deviation = 3.35); 21.14 in Study 3 (standard deviation = 4.30); and 20.08 in Study 4a (standard deviation = 4.53). The creativity of the idea submitted by a participant is measured as the average creativity rating across judges. Given each idea (target) was rated by a different set of judges, inter-judge reliability was assessed by computing an IntraClass Correlation (ICC) using a one-way random effects model [35], and ICC(1,k)=0.76 in Study 1, 0.78 in Study 2, 0.75 in Study 3, and 0.73 in Study 4a (Study 4b did not include an idea generation task).

### Data analysis plan

All studies included basic language/attention checks and participants were excluded if they failed any. Participants in Studies 1-4a were also excluded if they submitted ideas that were clearly off topic and/or used offensive language (see *Idea Generation*). To avoid online participants in Studies 1 and 4a-b using AI tools to enter responses, the Taskmaster tool [37] was used to track the amount of time spent by participants on task vs. off task. We removed participants who spent more than 10% of their time off task. See SI for a sensitivity analysis of the results with alternative thresholds of 5% and 15%. Only participants with complete data on all relevant tasks were included in each analysis. The initial number of complete responses were as follows for studies 1, 2, 3, 4a, 4b: 400, 133, 128, 500, 401. The number of participants removed for failing the attention or language check was 1 for Study 1 and 0 for studies 2, 3, 4a, 4b. The number of participants excluded due to their ideas being clearly off topic and/or offensive was 7 for Study 1, 5 for Study 2, 3 for Study 3, 7 for Study 4a, and 0 for Study 4b (that study did not include an idea generation task). In Studies 1, 4a and 4b respectively, 72, 51 and 36 participants were excluded due to spending more than 10% of their time off task. In Study 3, one additional respondent was excluded due to not properly following the instructions to the DAT by having four entries that were not single words, and hence having fewer than 7 usable entries. This led to final sample size of N = 320 participants for Study 1, N = 128 for Study 2, and N = 122 for Study 3, N = 442 in Study 4, N = 365 in Study 4b, with complete and usable data on all tasks. Correlations were computed as Pearson correlations. The link between various explanatory variables and idea generation ratings was analysed using OLS. Significance thresholds of 0.01, 0.05 and 0.10 are reported, and corrections for multiple comparisons are included and described when relevant. All statistical analysis were performed in Python. See SI for descriptive statistics of all relevant variables in each study.

## Results

### Correlation with creativity score

All studies find that people who use less circuitous (i.e., more optimally sequenced) semantic paths in the SSPT generate more creative ideas (Study 1 r = −0.20, p < 0.01, adjusted-p < 0.01; Study 2 r = −0.20, p < 0.05, adjusted-p < 0.10; Study 3 r = −0.20, p < 0.05, adjusted-p = 0.15; Study 4a r = −0.09, p < 0.05, adjusted-p < 0.10). To adjust for multiple comparisons in each study, we use a non-parametric permutation test [38]. We generated a null distribution by permuting the creativity scores 10,000 times and calculating, in each random permutation, the highest correlation coefficient between the shuffled creativity score and the other relevant variables. The adjusted p-value for a given correlation is then the proportion of random permutations in which the absolute value of the maximum correlation is greater than or equal to the absolute value of the original correlation.

These correlations are sizable compared to traditional measures of creative abilities. To test whether the correlation between the target variable, creativity, and one variable (in this case circuitousness) is higher in magnitude than the correlation between the target variable and another variable (in this case performance on the Unusual Uses Test), we employed the specific Z-test for dependent, overlapping correlations proposed by [39]. In Study 1, for example, only performance on the Unusual Uses Test is more correlated with creativity (r = 0.26, p < 0.01, adjusted-p < 0.01), but not significantly so (p = 0.44) (though this may be driven, in part, by the fact that performance on the Unusual Uses Test is also a creativity rating of ideas, generated in response to a different prompt – finding unusual uses for a wire hanger). In Study 2, circuitousness is more or equally correlated with creativity than any other measure (the correlation with the letter fluency task is r = 0.21, p < 0.05, adjusted-p < 0.10, not significantly different in magnitude from the correlation between circuitousness and creativity, p = 0.96). In Study 3, the correlation with circuitousness (r = −0.20, p < 0.05, adjusted-p = 0.11) is similar to the correlation achieved by the letter fluency task (r = 0.26, p < 0.01, adjusted-p < 0.05) and the Unusual Uses Test (r = 0.26, p < 0.01, adjusted-p < 0.05), with none of these correlations being significantly different from each other in magnitude (all p’s > 0.6). In Study 4a, circuitousness achieves a correlation (r = −0.09, p < 0.05, adjusted-p < 0.10) that is similar to the correlation achieved by the crystallized intelligence task (r = 0.13, p < 0.01, adjusted-p < 0.05), and these two correlations are not statistically significantly different in magnitude (p = 0.61).

To provide a robust estimate of the relationship between circuitousness and creativity, we conducted a fixed-effects “mini meta-analysis” across studies 1-4a [40]. This internal meta-analysis allows for the synthesis of effect sizes across the four studies, providing an estimate that transcends the sampling error of any single experiment. We convert the correlation between circuitousness and creativity performance to a Fisher z-score in each study, calculate a weighted average z-score (weighting by N-3 to account for varying sample sizes), transform the average z-score back to a correlation, and calculate the corresponding p-value and 95% confidence interval. This meta-analytic approach provides a more precise estimate of the effect size by aggregating power across our entire participant pool. We find that the predicted correlation across studies is r = −0.15, with a p-value<0.01 and a 95% confidence interval of [−0.21,-0.09]. For comparison, this predicted correlation is comparable to that obtained by applying the same procedure to the Unusual Uses Test (r = 0.23, p < 0.01, 95% CI=[0.15,0,31]) or the letter fluency task (r = 0.17, p < 0.01, 95% CI=[0.09,0.25]), and larger than that obtained for the Remote Associates Task (r = 0.00, p = 0.98, 95% CI=[−0.08.0.08]).

Applying a correction for attenuation to estimate the true correlation between circuitousness and creativity – using the test-retest ICC of 0.446 for circuitousness and a weighted average (across studies) ICC of 0.748 for creativity – increases the correlation to −0.26 (95% CI=[−0.37,-0.16]). With this correction, circuitousness accounts for approximately 7% of the variance in creativity.

### Predicting creativity score

Next, we examine whether the circuitousness measure coming from the SSPT explains creativity above and beyond extant measures. To do so, we regress the creativity of the idea from the idea generation task on the existing measures, and then add circuitousness as an additional predictor.

We standardize all measures, so that coefficients may be interpreted as the effect associated with a one standard deviation increase in the corresponding measure on the judged creativity of the idea (on a five-point scale). Table 1 reports the parameter estimates as well as model fit. See SI for more detailed results, including 95% confidence intervals, p-values and Variance Inflation Factors (VIF).

**Table 1:** Predicting creativity of submitted idea.

	Study 1		Study 2		Study 3		Study 4	
Letter fluency: number of unique words	0.029	0.008	0.116*	0.108*	0.098^	0.092	--	--
Unusual uses test: judged creativity	0.130**	0.130**	0.043	0.044	0.105^	0.096^	--	--
Remote associates test: number of correct responses	−0.027	−0.042	−0.077	−0.094^	−0.053	−0.058	--	--
Divergent association task: pairwise distance	--	--	--	--	−0.018	−0.018	--	--
Forward flow: pairwise distance	--	--	--	--	0.026	0.025	--	--
Crystallized intelligence: number of correct responses	--	--	--	--	--	--	0.067**	0.062*
Shortest semantic path task: circuitousness	--	−0.097**	--	−0.105*	--	−0.080^	--	−0.042^
Number of parameters	4	5	4	5	6	7	2	3
Number of observations	320	320	128	128	122	122	442	442
F-statistic	8.19	9.16	2.858	3.586	2.596	2.674	7.623	5.288
R^2^	0.072	0.104	0.065	0.104	0.101	0.122	0.017	0.024
Adjusted R^2^	0.063	0.093	0.042	0.075	0.062	0.077	0.015	0.019

*:p < 0.05. **: p < 0.01. ^:p < 0.1.

Results indicate that, even after controlling for the other measures, circuitousness adds predictive power. Further, its inclusion leads to substantial improvements in the model’s adjusted R^2^ (i.e., + 48% or 0.030 in Study 1, 79% or 0.033 in Study 2, 24% or 0.015 in Study 3, + 27% or 0.004 in Study 4a).

### Correlation with other tasks

Results so far suggest that circuitousness predicts creativity as well as (or better than) prior measures and is able to pick up novel aspects. To better understand how circuitousness relates to extant measures, Table 2 illustrates the correlations with the other tasks. We again adjust for multiple comparisons in each study using a non-parametric permutation test ([38]), using this time circuitousness as the target variable. Results indicate that circuitousness is somewhat, but not strongly, related to standard measures of divergent thinking (e.g., the Unusual Use Test, the Divergent Association Task and the Forward Flow Task). While circuitousness is correlated with the creativity rating of the single (typically more complex and elaborate) idea submitted by participants, it is not as strongly related to the creativity rating of the set of simple ideas submitted by participants in the Unusual Uses Test. Circuitousness is also somewhat, but not strongly, related to performance at the letter fluency task or the crystallized intelligence task.

**Table 2:** Correlation between circuitousness of the shortest semantic path and other measures.

	Study 1	Study 2	Study 3	Study 4a
Unusual uses test: judged creativity	−0.11^^,ns^	−0.08 ^ns,ns^	−0.18*^,ns^	n/a
Divergent association task: pairwise distance	n/a	n/a	−0.05 ^ns,ns^	n/a
Forward flow: pairwise distance	n/a	n/a	−0.04 ^ns,ns^	n/a
Letter fluency: number of unique words	−0.28**^,^**	−0.13 ^ns,ns^	−0.16^^,ns^	n/a
Remote associates test: number of correct responses	−0.23**^,^**	−0.18*^,ns^	−0.13 ^ns,ns^	n/a
Crystallized intelligence: number of correct responses	n/a	n/a	n/a	−0.11*^,^*
Idea generation task: judged creativity	−0.20**^,^**	−0.20*^,^^	−0.20*^,ns^	−0.09*^,^^

Unadjusted and adjusted p-values (using permutation correction). *:p < 0.05. **: p < 0.01. ^: p < 0.10. ns: p > 0.10.

Circuitousness is also somewhat related with performance on the Remote Associates Test, such that lower circuitousness (more efficient semantic search) is associated with stronger performance. The Remote Associates Test is typically considered a convergent thinking test. While it does not require sequential semantic search and it only asks participants to retrieve a single concept, finding the correct answer on this task does require performing an efficient semantic search under constraint, and it is possible that this commonality explains the correlation with our measure of circuitousness. However, as discussed above, our data suggests that circuitousness is a stronger predictor of the creativity of generated ideas.

### Circuitousness vs. Path Length

We argued that circuitousness, which measures the efficient sequencing of concepts along a semantic path, is distinct from the length of the semantic path retrieved, and that only the former should correlate with creative performance. To test this, we also compute Path Length: the sum of Euclidean distances between each word in the sequence and the next one, *d*_*t*_ = ||*w*_*t+1*_-*w*_*t*_||, divided by the distance ||*w*_*5*_-*w*_*1*_|| which serves as a theoretical minimal path length. We standardize Path Length and average it across rounds, as we do for Circuitousness.

Exploratory, non pre-registered analyses find that the correlation between Circuitousness and Path Length is only moderate (r = 0.21 in Study 1, r = 0.13 in Study 2, r = 0.15 in Study 3, r = 0.08 in Study 4a, r = 0.31 in Study 4b), and that Path Length is not significantly correlated with the creativity score (r = −0.02 in Study 1, r = −0.06 in Study 2, r = −0.01 in Study 3, r = 0.05 in Study 4a, all p’s > 0.32). This confirms that Circuitousness and Path Length are distinct concepts, both theoretically and empirically, and that only the former is predictive of creative performance.

### Psychometric Properties

#### Test-rest reliability.

We explore test-retest reliability using Studies 4a-b, which administered the same tasks in the same order for each participant. After applying our pre-registered exclusion criteria (based on time spent off task), there is a sample of N = 338 respondents who completed the same 10 SSPT items in the same order, one month apart. The Pearson correlation between circuitousness in Study 4a vs. Study 4b is 0.448 (95% CI=[0.358,0.529]) and the IntraClass Correlation ICC(3,1) [35] is 0.446 (95% CI=[0.360,0.530]). As a comparison, [10] find a test-retest reliability for Forward Flow of r = 0.40, and [11] report a test-retest reliability for the DAT of r = 0.73.

#### Internal reliability and construct validity.

Because our measure of circuitousness is based on multiple tasks, we can also explore internal reliability and construct validity (Forward Flow and the DAT do not lend themselves to such analysis). We calculate Cronbach’s alpha between SSPT tasks in each study (where a task is defined by a pair of seed words). It is 0.431 in Study 1 (95% CI: [0.315,0.533]); 0.348 in Study 2 (95% CI: [0.112,0.536]); 0.147 in Study 3 (95% CI: [−0.177,0.404]); 0.333 in Study 4a (95% CI: [0.215,0.441]); 0.547 in Study 4b (95% CI: [0.458,0.626]). (See SI for the correlations between tasks in each study.) In studies 4a-b, which each included two sets of five tasks, we also explore internal reliability by measuring split-half reliability. In Study 4a (4b) we find a raw Spearman correlation in the average standardized circuitousness between the two sets of five tasks of 0.23 (0.30) and a Spearman-Brown corrected reliability of 0.37 (0.47). We also perform a factor analysis to explore the extent to which circuitousness is a unitary construct. Scree plots are reported in the SI. We find a McDonald’s Omega of 0.476 in Study 1, 0.356 in Study 2, 0.363 in Study 3, 0.338 in Study 4a, 0.549 in Study 4b. This means that only about 30% to 50% of the variance in our circuitousness score is due to a common factor.

Overall, these exploratory analyses suggest that the current version of the SSPT, despite its reasonable discriminant and predictive validity, has moderate-to-low psychometric properties. However, as mentioned above the test-retest reliability of circuitousness that we find is slightly higher than that reported by [10] for Forward Flow, a well-established predictor of creativity. More generally, these results should be interpreted in the context of mounting evidence across fields showing that many cognitive task measures have moderate-to-low reliability [41–44], for example, report test-retest reliabilities for seven classic cognitive tasks (including Flanker task and the Stroop task) ranging from 0 to 0.82, “surprisingly low for most tasks given their common use” (p 1166), with most tasks having reliability below the standard cut-off of 0.7 and some having reliabilities below 0.4 (while ours is above 0.4). In a related cognitive context, in their large-scale analysis of test-retest reliabilities of self-regulation measures, [45] find that behavioral tasks measuring self-regulation have an average test-retest reliability (ICC) of 0.331 (lower than the value we find), which is significantly lower than the test-retest reliability of self-reported measures. Along these lines, [44, 45] suggest that low test-retest reliability does not invalidate the existence of an instrument, but rather undermines its suitability as a *trait*. Regardless, our studies suggest that optimal sequencing of concepts during semantic search (quantified using circuitousness), measured using the tasks we developed here (SSPT), significantly correlates with the creativity rating of ideas generated by participant in the same session. Interestingly, circuitousness measured in Study 4b is also significantly correlated with creativity ratings from Study 4a (r = −0.147, p < 0.01), suggesting that correlation holds across, and not only within, session.

## Discussion

Creativity is associated with the efficient (i.e., limited cognitive effort) retrieval of relevant and useful concepts. Accordingly, creative ability has been associated with memory structures that allow individuals to move effortlessly around semantic spaces in search of solutions to a problem. This has led to the development of several tasks that measure divergent thinking.

But while having a memory structure that retrieves more distant concepts with less effort is important, retrieving concepts in an optimal sequence should also be relevant, allowing one to cover more semantic ground per unit of effort. In other words, the retrieval of relevant concepts should be enhanced both by moving effortlessly in the space, as well as by spending cognitive effort wisely and efficiently by avoiding repetitions or other unnecessarily long steps. Based on this reasoning, we propose a new task (SSPT) and associated measure (circuitousness) that measures optimal sequencing when retrieving semantic paths. Initial validation studies demonstrate that this measure adds to the ability to predict creative performance. Participants’ performance at the SSPT (measured by circuitousness) is robustly associated with their creativity while being distinct from traditional measures of creative abilities. That said, our conclusions are preliminary and our initial studies present limitations which could be addressed in future research.

First and foremost, the reported psychometric properties are modest to low. The consistent correlation we find between circuitousness and creativity is potentially attenuated by the low reliability of the circuitousness measure. Hence, the fact that our initial implementation of the task significantly and robustly predicts creativity, even while controlling for a host of traditional measures, suggests that future improvements and refinements of the task and its associated measure of circuitousness have the potential to unlock significant theoretical and empirical advances in the study of the creative process. The correlation we find between circuitousness and creativity may viewed as a lower bound of what may be achieved with refined versions of the task and associated measure. One reason internal reliability might be attenuated is that participants are learning across multiple rounds (and improving their performance), but ancillary analyses suggest this is unlikely. We explore the evolution of circuitousness across rounds (i.e., we consider the standardized circuitousness in the task completed in first position, second position, etc., where standardization is still performed separately for each pair of seed words) in Studies 4a-b (the random order in which tasks were shown was not recorded in Studies 1–3), but there is no evidence of learning across the 10 rounds (see SI).

Another potential explanation for the low internal reliability may be that each pair of seed words taps into different areas of the semantic space, which may be more or less familiar to the participant (similar to how trivia questions might test knowledge in different areas with which participants may be more or less familiar). If that is the case, future research might select seed words that are more directly relevant to the domain in which creativity is being measured (e.g., selecting health-related seed words if the target creativity task asks for health-related ideas). However, the moderate test-retest reliability suggests that that the low internal reliability and construct validity is probably not entirely due to this. Indeed, if each pair of seed words testing a different subarea of the semantic space were the main issue, the correlation should be improved when administering the same tasks (with the same sets of seed words) twice.

Yet another potential explanation is that performance at the SSPT is just noisy and influenced by many random factors, similar to how the speed with which someone completes a maze may be influenced by random factors such as the first steps they take. But if our measure of circuitousness were truly “just noise,” we would not find a significant, directional correlation across 4 independent studies.

Nevertheless, future research should attempt to improve the reliability of the SSPT and associated circuitousness measure, perhaps using the strategies laid out by [46] aiming at increasing between-participant variability and/or decreasing measurement error.

A second limitation, beyond the reported psychometric properties, is that our studies did not include demographic data. Future research might explore systematic variations across demographic groups. Third, in our studies the participants who provided creativity ratings were not trained beyond basic instructions, they did not receive examples of creative vs. non-creative responses, and there was no calibration process among raters. The absence of these procedures may have introduced systematic variability in creativity scores, and future studies should consider more standardized evaluation protocols. Fourth, we only tested one idea generation task (health app ideas) and one type of semantic sequencing task (connecting 2 words with 3 intermediates). Future research may explore whether our findings generalize to: (a) other types of creative tasks (artistic, scientific, problem-solving), (b) real-world creativity, (c) different SSPT formats, and (d) other semantic domains. Fifth, we cannot rule out that circuitousness is a measure of executive control that is not specific to creativity. Future research may explore whether circuitousness also predicts performance at other tasks beyond creativity.

## Supporting information

S1 Text Supplemental Information for “Optimally Sequencing Semantic Search Predicts Creativity”. (DOCX)
