# Supplementary material for: Optimally sequencing semantic search predicts creativity
Source: PLoS One. 2026 Jun 24;21(6):e0352328. doi: 10.1371/journal.pone.0352328 (PMC13293437; doi:10.1371/journal.pone.0352328)

Supplemental Information for “Optimally Sequencing Semantic Search Predicts Creativity”

**List of Words in Crystallized Intelligence Test**

The target name is listed in upper case font, followed by the choice options in parentheses, with the correct answer in *italics*.

*Synonyms:*

CONCUR (*acquiesce*, extricate, divulge, concoct, ransack)

CONFISCATE (harass, repulse, console, *appropriate*, congregate)

SOLICIT (purge, spurn, entrance, exert, *beseech*)

FURTIVE (ecstatic, heinous, *stealthy*, flimsy, facile)

ASTUTE (bizarre, ascetic, *sagacious*, lineal, irritable)

COVET (*crave*, claim, avenge, clutch, comply)

OSCILLATE (premeditate, irradiate, *vacillate*, recapitulate, furbish)

INDOLENT (contrite, inexhaustible, impervious, arduous, *slothful*)

DISPARITY (despondency, mediocrity, serenity, *incongruity*, assiduity)

INDIGENT (refractory, fiscal, *destitute*, tolerable, diligent)

*Antonyms:*

SATED (*famished*, finished, finicky, fulfilled, fortunate)

COMPLAISANT (distasteful, egoistical, alone, ugly, *recalcitrant*)

ALOOF (happy, deadly, *gregarious*, manly, varied)

ABOMINATE (*adore*, despair, abate, deplore, attach)

VERBOSE (garrulous, magnificent, grandiloquent, *taciturn*, calculating)

DEARTH (birth, brevity, *abundance*, splendor, renaissance)

CORPULENT (sallow, affiliated, *emaciated*, entrepreneur, anemic)

GERMANE (teutonic, healthful, *irrelevant*, massive, puny)

VACUOUS (bankrupt, loose, livid, superficial, *profound*)

SPORADIC (germinal, antiseptic, *incessant*, summery, wintry)

**Descriptive Statistics**

Study 1:

|  | N | Mean | Std. Dev | Min | Median | Max |
| --- | --- | --- | --- | --- | --- | --- |
| Circuitousness | 320 | 0.011 | 0.584 | -0.513 | -0.145 | 4.169 |
| Idea generation task: judged creativity | 320 | 3.276 | 0.516 | 1.800 | 3.250 | 4.348 |
| Letter fluency: number of unique words | 320 | 15.250 | 5.922 | 1 | 16 | 29 |
| Unusual uses test: judged creativity | 320 | 3.273 | 0.760 | 1.053 | 3.316 | 4.875 |
| Remote Associates test: number of correct responses | 320 | 2.581 | 1.537 | 0 | 3 | 7 |

Study 2:

|  | N | Mean | Std. Dev | Min | Median | Max |
| --- | --- | --- | --- | --- | --- | --- |
| Circuitousness | 128 | 0.002 | 0.549 | -0.584 | -0.151 | 2.756 |
| Idea generation task: judged creativity | 128 | 3.208 | 0.518 | 1.417 | 3.282 | 4.105 |
| Letter fluency: number of unique words | 128 | 17.969 | 6.264 | 1 | 19 | 36 |
| Unusual uses test: judged creativity | 128 | 3.116 | 0.852 | 1.000 | 3.269 | 4.550 |
| Remote Associates test: number of correct responses | 128 | 2.047 | 1.441 | 0 | 2 | 6 |

Study 3:

|  | N | Mean | Std. Dev | Min | Median | Max |
| --- | --- | --- | --- | --- | --- | --- |
| Circuitousness | 122 | 0.014 | 0.519 | -0.535 | -0.163 | 1.828 |
| Idea generation task: judged creativity | 122 | 3.222 | 0.529 | 1.438 | 3.200 | 4.429 |
| Letter fluency: number of unique words | 122 | 15.762 | 5.573 | 2 | 16 | 29 |
| Unusual uses test: judged creativity | 122 | 3.252 | 0.755 | 1.385 | 3.421 | 4.833 |
| Remote Associates test: number of correct responses | 122 | 1.943 | 1.281 | 0 | 2 | 5 |
| Divergent association task: pairwise distance | 122 | 0.882 | 0.042 | 0.663 | 0.889 | 0.957 |
| Forward flow: pairwise distance | 122 | 0.823 | 0.070 | 0.557 | 0.841 | 0.927 |

Study 4a:

|  | N | Mean | Std. Dev | Min | Median | Max |
| --- | --- | --- | --- | --- | --- | --- |
| Circuitousness | 442 | 0.008 | 0.408 | -0.488 | -0.075 | 1.832 |
| Idea generation task: judged creativity | 442 | 3.169 | 0.513 | 1.733 | 3.170 | 4.409 |
| Crystallized intelligence: number of correct responses | 442 | 12.523 | 4.909 | 1 | 13 | 20 |

Study 4b:

|  | N | Mean | Std. Dev | Min | Median | Max |
| --- | --- | --- | --- | --- | --- | --- |
| Circuitousness | 365 | 0.004 | 0.438 | -0.502 | -0.055 | 3.121 |

**Full Regression Results**

Study 1:

|  | Coefficient | 95% CI | p-value | VIF |
| --- | --- | --- | --- | --- |
| Letter fluency: number of unique words | 0.008 | [-0.054, 0.069] | 0.806 | 1.293 |
| Unusual uses test: judged creativity | 0.130** | [0.072, 0.187] | 0.000 | 1.126 |
| Remote associates test: number of correct responses | -0.042 | [-0.100, 0.017] | 0.159 | 1.167 |
| Shortest semantic path task: circuitousness | -0.097** | [-0.154, -0.040] | 0.001 | 1.109 |
| Number of observations | 320 |  |  |  |
| F-Statistic | 9.164 |  |  |  |
| R-Squared | 0.104 |  |  |  |
| Adj. R-Squared | 0.093 |  |  |  |

Study 2:

|  | Coefficient | 95% CI | p-value | VIF |
| --- | --- | --- | --- | --- |
| Letter fluency: number of unique words | 0.108* | [0.009, 0.206] | 0.032 | 1.267 |
| Unusual uses test: judged creativity | 0.044 | [-0.055, 0.143] | 0.382 | 1.284 |
| Remote associates test: number of correct responses | -0.094^ | [-0.191, 0.002] | 0.056 | 1.218 |
| Shortest semantic path task: circuitousness | -0.105* | [-0.194, -0.016] | 0.021 | 1.041 |
| Number of observations | 128 |  |  |  |
| F-Statistic | 3.586 |  |  |  |
| R-Squared | 0.104 |  |  |  |
| Adj. R-Squared | 0.075 |  |  |  |

Study 3:

|  | Coefficient | 95% CI | p-value | VIF |
| --- | --- | --- | --- | --- |
| Letter fluency: number of unique words | 0.092 | [-0.019, 0.203] | 0.105 | 1.47 |
| Unusual uses test: judged creativity | 0.096^ | [-0.017, 0.209] | 0.094 | 1.513 |
| Remote associates test: number of correct responses | -0.058 | [-0.158, 0.041] | 0.248 | 1.178 |
| Divergent association task: pairwise distance | -0.018 | [-0.116, 0.079] | 0.709 | 1.127 |
| Forward flow: pairwise distance | 0.025 | [-0.071, 0.121] | 0.603 | 1.095 |
| Shortest semantic path task: circuitousness | -0.080^ | [-0.174, 0.014] | 0.094 | 1.045 |
| Number of observations | 122 |  |  |  |
| F-Statistic | 2.674 |  |  |  |
| R-Squared | 0.122 |  |  |  |
| Adj. R-Squared | 0.077 |  |  |  |

Study 4a:

|  | Coefficient | 95% CI | p-value | VIF |
| --- | --- | --- | --- | --- |
| Crystallized intelligence: number of correct responses | 0.062* | [0.015, 0.110] | 0.011 | 1.012 |
| Shortest semantic path task: circuitousness | -0.042^ | [-0.090, 0.006] | 0.088 | 1.012 |
| Number of observations | 442 |  |  |  |
| F-Statistic | 5.288 |  |  |  |
| R-Squared | 0.024 |  |  |  |
| Adj. R-Squared | 0.019 |  |  |  |

**Correlation Between SSPT Tasks and Between Each Task and Creativity**

We report the correlation between circuitousness in each round of the SSPT and the other rounds, as well as creativity.

Study 1:

|  | Task 2 | Task 3 | Task 4 | Task 5 | Idea generation task: judged creativity |
| --- | --- | --- | --- | --- | --- |
| Task 1 | -0.007 | 0.178 | 0.175 | 0.099 | -0.015 |
| Task 2 |  | -0.043 | 0.053 | 0.116 | -0.002 |
| Task 3 |  |  | 0.255 | 0.142 | -0.080 |
| Task 4 |  |  |  | 0.369 | -0.128 |
| Task 5 |  |  |  |  | -0.132 |

Study 2:

|  | Task 2 | Task 3 | Task 4 | Task 5 | Idea generation task: judged creativity |
| --- | --- | --- | --- | --- | --- |
| Task 1 | 0.035 | 0.175 | 0.025 | 0.029 | -0.131 |
| Task 2 |  | 0.135 | 0.036 | 0.029 | -0.012 |
| Task 3 |  |  | 0.170 | 0.097 | -0.133 |
| Task 4 |  |  |  | 0.253 | -0.203 |
| Task 5 |  |  |  |  | -0.035 |

Study 3:

|  | Task 2 | Task 3 | Task 4 | Task 5 | Idea generation task: judged creativity |
| --- | --- | --- | --- | --- | --- |
| Task 1 | -0.008 | 0.046 | 0.072 | 0.199 | -0.131 |
| Task 2 |  | 0.043 | -0.028 | -0.002 | -0.165 |
| Task 3 |  |  | -0.113 | -0.011 | -0.002 |
| Task 4 |  |  |  | 0.185 | -0.040 |
| Task 5 |  |  |  |  | -0.074 |

Study 4a:

|  | # 2 | # 3 | # 4 | # 5 | #6 | #7 | #8 | #9 | #10 | creativity |
| --- | --- | --- | --- | --- | --- | --- | --- | --- | --- | --- |
| # 1 | 0.037 | 0.081 | 0.060 | 0.010 | 0.070 | 0.080 | 0.095 | 0.048 | 0.086 | 0.007 |
| # 2 |  | 0.054 | -0.004 | 0.053 | 0.038 | -0.058 | 0.095 | 0.232 | 0.050 | -0.072 |
| # 3 |  |  | 0.059 | -0.006 | 0.119 | 0.070 | 0.063 | -0.011 | -0.008 | -0.006 |
| # 4 |  |  |  | -0.026 | 0.140 | -0.022 | 0.096 | 0.016 | 0.193 | -0.011 |
| # 5 |  |  |  |  | -0.050 | -0.028 | 0.105 | 0.067 | 0.059 | -0.024 |
| # 6 |  |  |  |  |  | 0.101 | 0.081 | -0.015 | 0.071 | -0.013 |
| # 7 |  |  |  |  |  |  | 0.081 | 0.046 | 0.034 | 0.069 |
| # 8 |  |  |  |  |  |  |  | 0.027 | 0.201 | -0.103 |
| # 9 |  |  |  |  |  |  |  |  | 0.073 | -0.134 |
| # 10 |  |  |  |  |  |  |  |  |  | -0.125 |

Study 4b (no creativity rating collected):

|  | # 2 | # 3 | # 4 | # 5 | #6 | #7 | #8 | #9 | #10 |
| --- | --- | --- | --- | --- | --- | --- | --- | --- | --- |
| # 1 | 0.004 | 0.083 | 0.158 | 0.108 | 0.124 | -0.039 | 0.227 | 0.121 | 0.135 |
| # 2 |  | 0.023 | 0.074 | 0.062 | -0.001 | 0.031 | 0.041 | 0.046 | -0.014 |
| # 3 |  |  | 0.076 | 0.202 | 0.041 | -0.037 | 0.144 | 0.139 | 0.197 |
| # 4 |  |  |  | 0.155 | 0.102 | 0.123 | 0.254 | 0.087 | 0.112 |
| # 5 |  |  |  |  | 0.070 | -0.021 | 0.158 | 0.027 | 0.170 |
| # 6 |  |  |  |  |  | -0.003 | 0.143 | 0.106 | 0.165 |
| # 7 |  |  |  |  |  |  | 0.035 | 0.150 | -0.015 |
| # 8 |  |  |  |  |  |  |  | 0.117 | 0.215 |
| # 9 |  |  |  |  |  |  |  |  | 0.070 |

**Scree Plots from Factor Analysis**

Study 1:


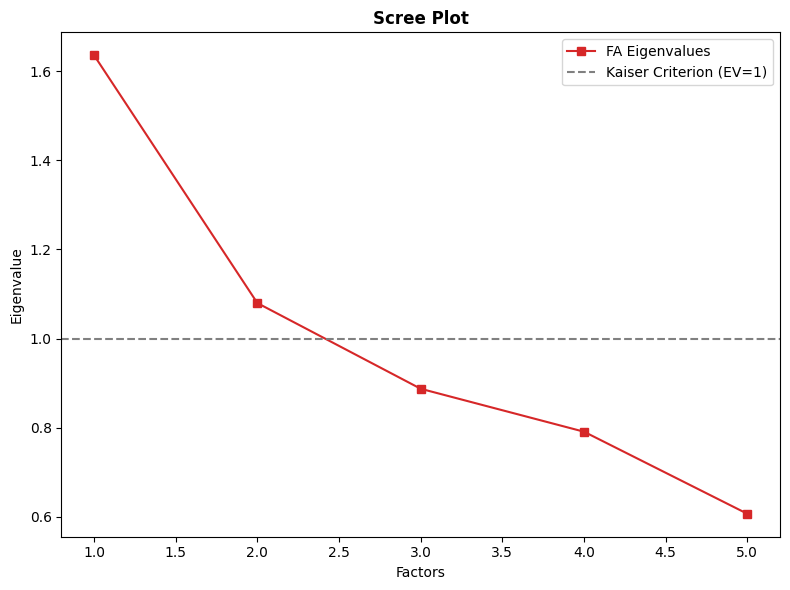


Study 2:


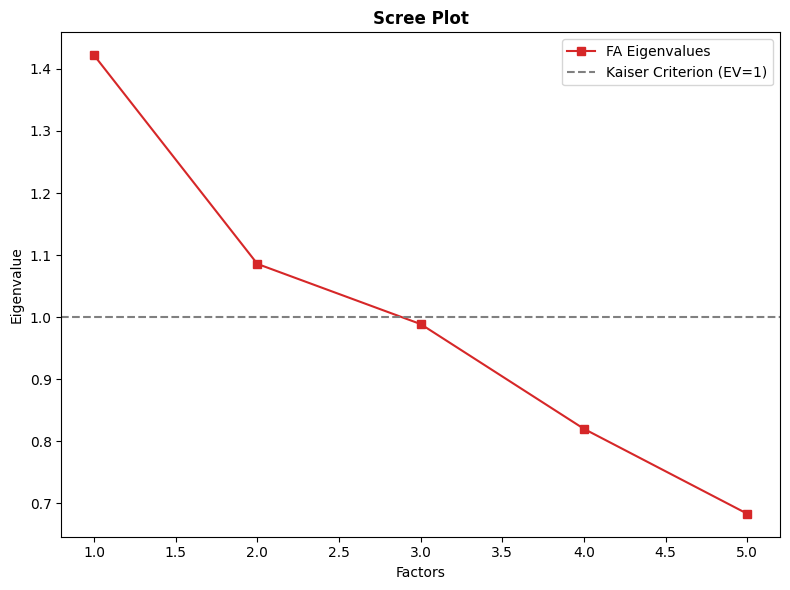


Study 3:


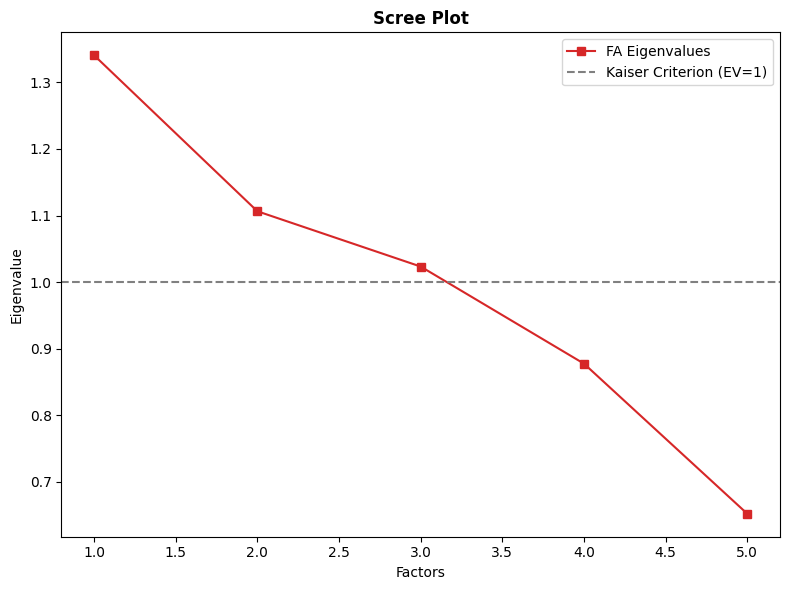


Study 4a:


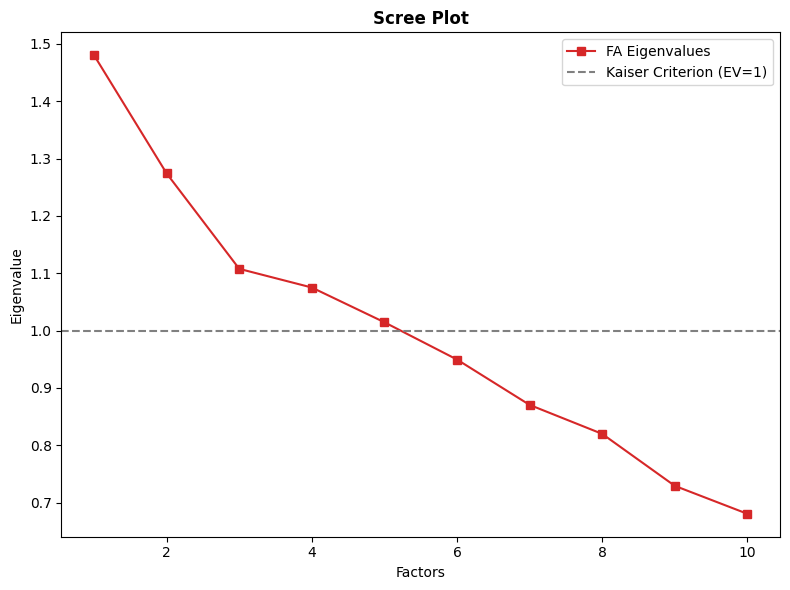


Study 4b:


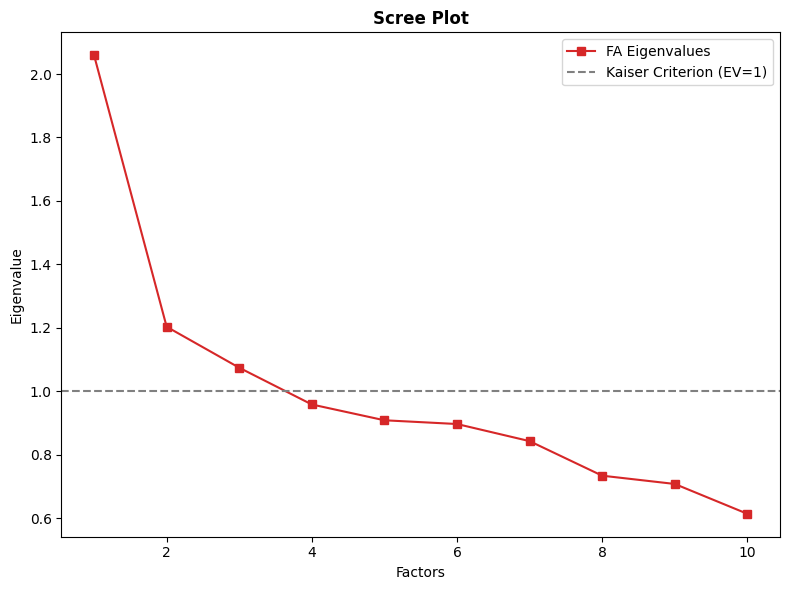


**SSPT Performance Evolution Across Tasks**

Study 4a:


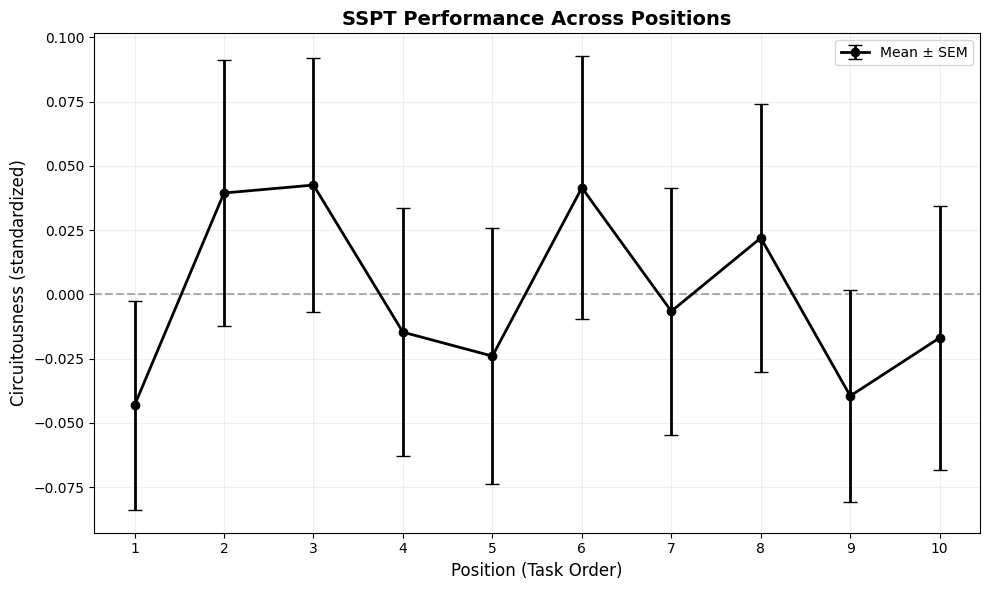


Study 4b:


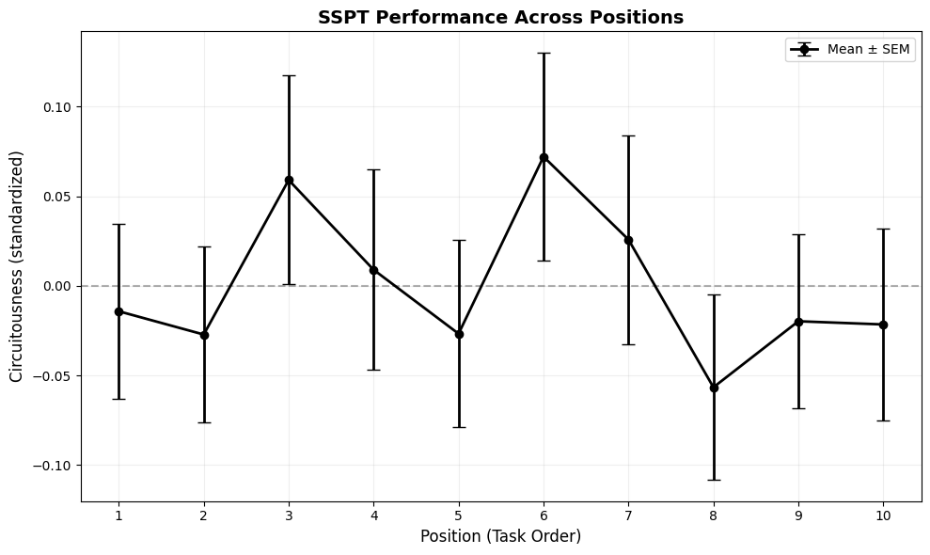


**Robustness to Exclusion Criteria in Online Studies (1, 4a,4b)**

Study 1:

Predicting creativity of submitted idea.

|  | 5% threshold | | 15% threshold | |
| --- | --- | --- | --- | --- |
| Letter fluency: number of unique words | 0.033 | 0.011 | 0.030 | 0.008 |
| Unusual uses test: judged creativity | 0.126** | 0.127** | 0.130** | 0.129** |
| Remote associates test: number of correct responses | -0.039 | -0.052^ | -0.029 | -0.044 |
| Shortest semantic path task: circuitousness | -- | -0.096** | -- | -0.098** |
| Number of parameters | 4 | 5 | 4 | 5 |
| Number of observations | 298 | 298 | 331 | 331 |
| R^2^ | 0.071 | 0.102 | 0.074 | 0.107 |
| Adjusted R^2^ | 0.062 | 0.090 | 0.065 | 0.096 |

*:p<0.05. **: p<0.01. ^:p<0.1.

Correlation between circuitousness of the shortest semantic path and other measures.

|  | 5% threshold | 15% threshold |
| --- | --- | --- |
| Unusual uses test: judged creativity | -0.08^ns,ns^ | -0.12^ns,ns^ |
| Letter fluency: number of unique words | -0.27^**,**^ | -0.29^**,**^ |
| Remote associates test: number of correct responses | -0.20^**,**^ | -0.24^**,**^ |
| Idea generation task: judged creativity | -0.19^**,**^ | -0.20^**,**^ |

Unadjusted and adjusted p-values (using permutation correction). *:p<0.05. **: p<0.01. ^: p<0.10. ns: p>0.10.

Study 4a:

Predicting creativity of submitted idea.

|  | 5% threshold | | 15% threshold | |
| --- | --- | --- | --- | --- |
| Crystallized intelligence: number of correct responses | 0.072** | 0.066** | 0.069** | 0.064** |
| Shortest semantic path task: circuitousness | -- | -0.047^ | -- | -0.040^ |
| Number of parameters | 2 | 2 | 2 | 3 |
| Number of observations | 434 | 434 | 451 | 451 |
| R^2^ | 0.019 | 0.028 | 0.018 | 0.024 |
| Adjusted R^2^ | 0.017 | 0.023 | 0.015 | 0.019 |

*:p<0.05. **: p<0.01. ^:p<0.1.

Correlation between circuitousness of the shortest semantic path and other measures.

|  | 5% threshold | 15% threshold |
| --- | --- | --- |
| Crystallized intelligence | -0.11*^,^^ | -0.11*^,^* |
| Idea generation task: judged creativity | -0.11*^,^^ | -0.09^^,ns^ |

Unadjusted and adjusted p-values (using permutation correction). *:p<0.05. **: p<0.01. ^: p<0.10. ns: p>0.10.

**Robustness to Controlling for Number of Uses in Unusual Uses Test**

Predicting creativity of submitted idea

|  | Study 1 | | Study 2 | | Study 3 | |
| --- | --- | --- | --- | --- | --- | --- |
| Letter fluency: number of unique words | 0.021 | 0.001 | 0.148** | 0.140** | 0.093 | 0.082 |
| Unusual uses test: number of uses | 0.053 | 0.047 | -0.183** | -0.193** | 0.025 | 0.046 |
| Unusual uses test: judged creativity | 0.097* | 0.101** | 0.153* | 0.160** | 0.095 | 0.076 |
| Remote associates test: number of correct responses | -0.027 | -0.042 | -0.069 | -0.086^ | -0.053 | -0.059 |
| Divergent association task: pairwise distance | -- | -- | -- | -- | -0.019 | -0.021 |
| Forward flow: pairwise distance | -- | -- | -- | -- | 0.023 | 0.019 |
| Shortest semantic path task: circuitousness | -- | -0.096** | -- | -0.113* | -- | -0.087^ |
| Number of parameters | 5 | 6 | 5 | 6 | 7 | 8 |
| Number of observations | 320 | 320 | 128 | 128 | 122 | 122 |
| F-statistic | 6.622 | 9.16 | 4.459 | 5.094 | 2.179 | 2.372 |
| R^2^ | 0.078 | 0.104 | 0.127 | 0.173 | 0.102 | 0.127 |
| Adjusted R^2^ | 0.066 | 0.093 | 0.098 | 0.139 | 0.055 | 0.074 |

**Screenshots from all Tasks**

*Shortest Semantic Path Task.*

General instructions:


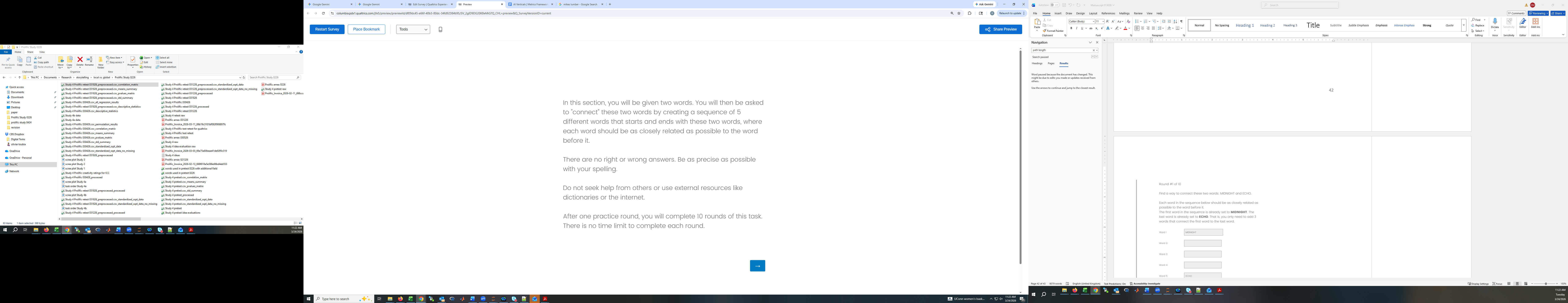


Task-level instructions:


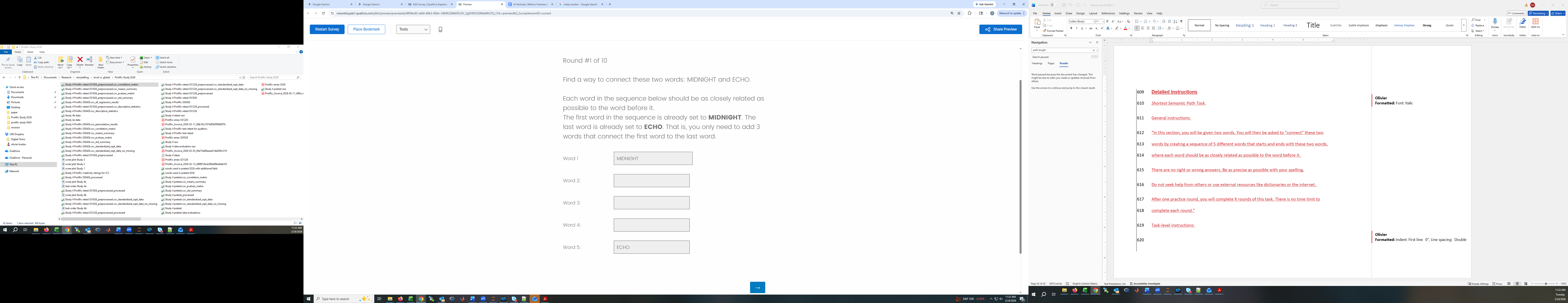


*Letter Fluency Task:*

General instructions:


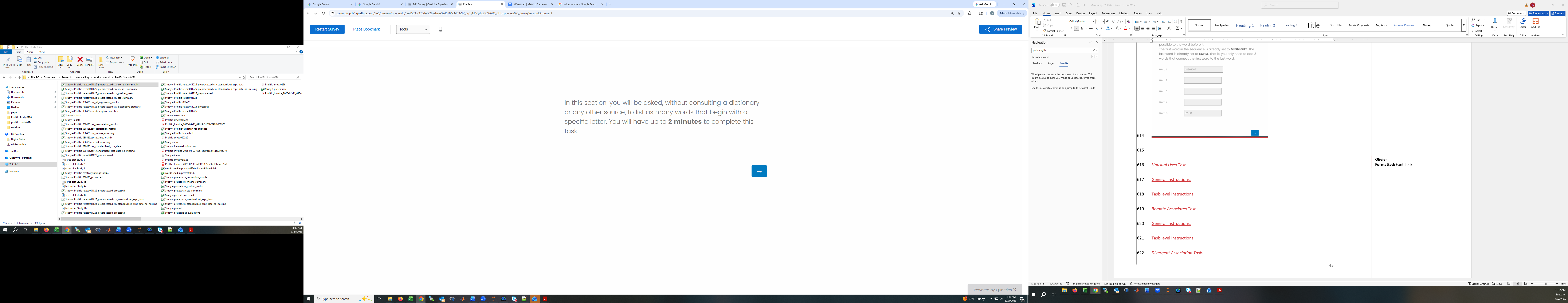


Task-level instructions:


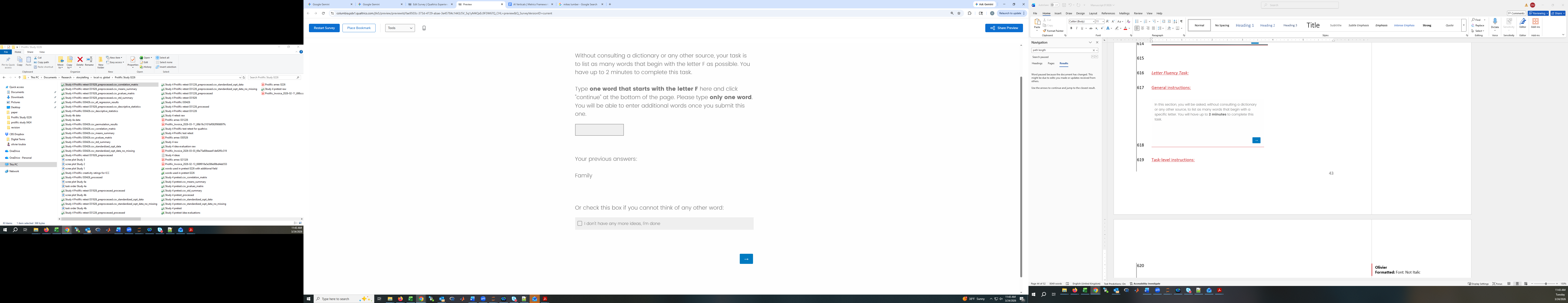


*Unusual Uses Test.*

General instructions:


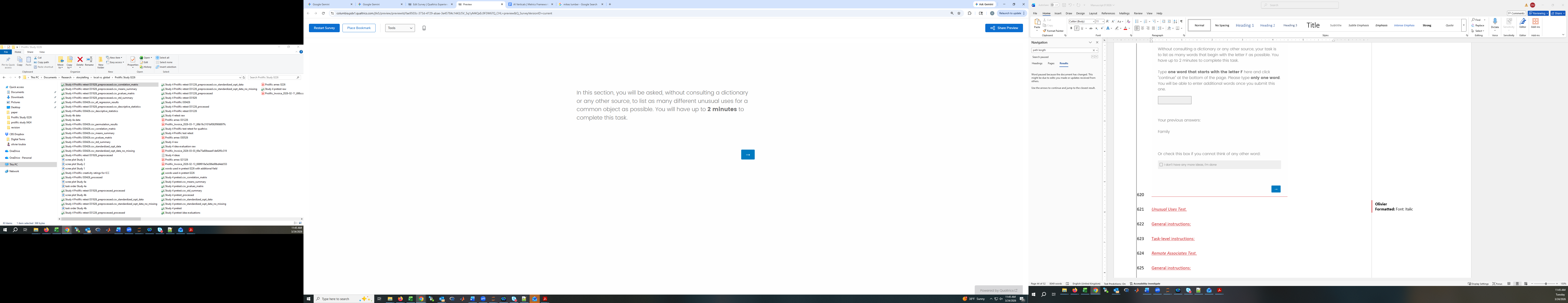


Task-level instructions:


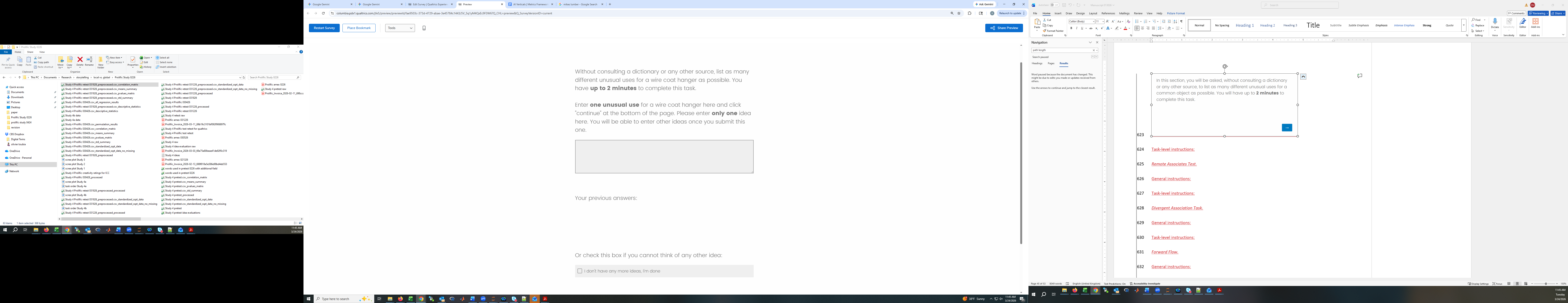


*Remote Associates Test.*

General instructions:


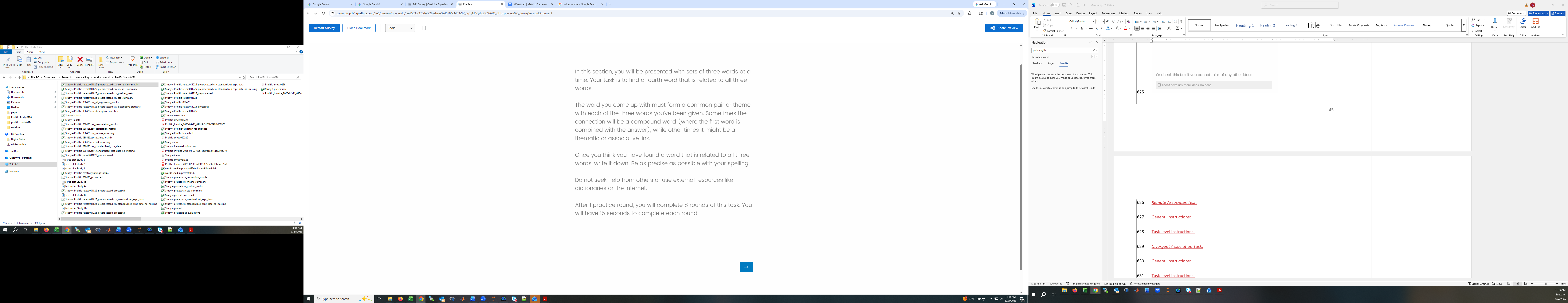


Task-level instructions:


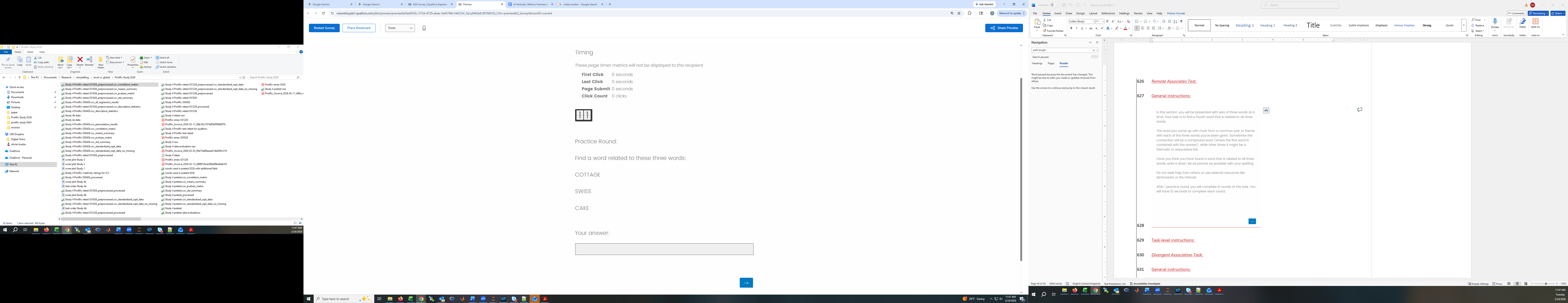


*Divergent Association Task.*

General instructions:


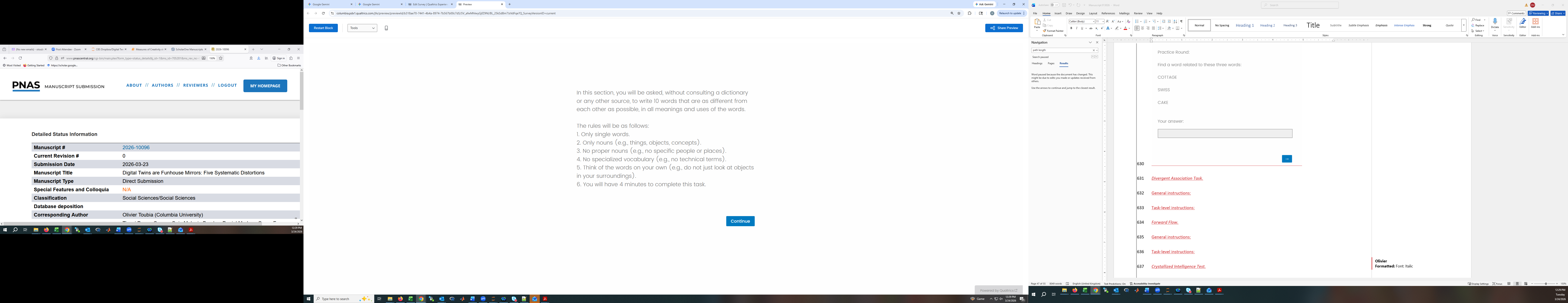


Task-level instructions:


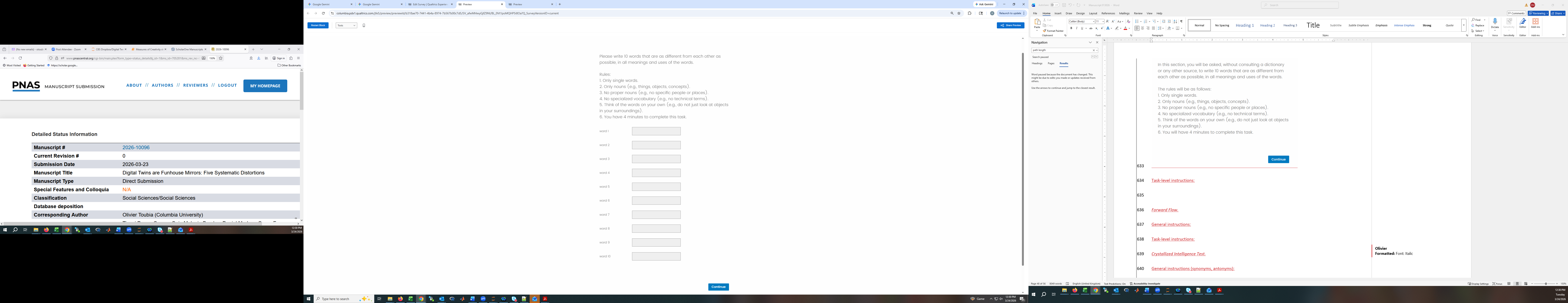


*Forward Flow* *Instructions*.


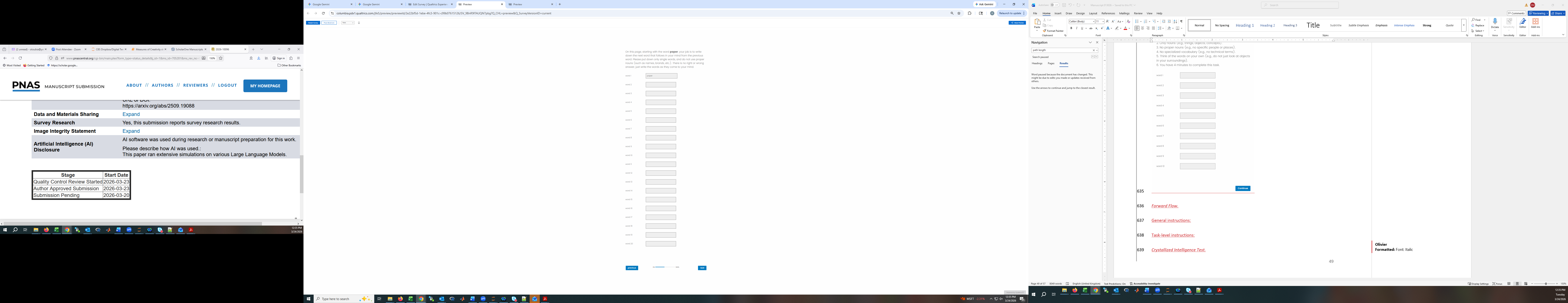


*Crystallized Intelligence Test.*

General instructions (synonyms, antonyms):


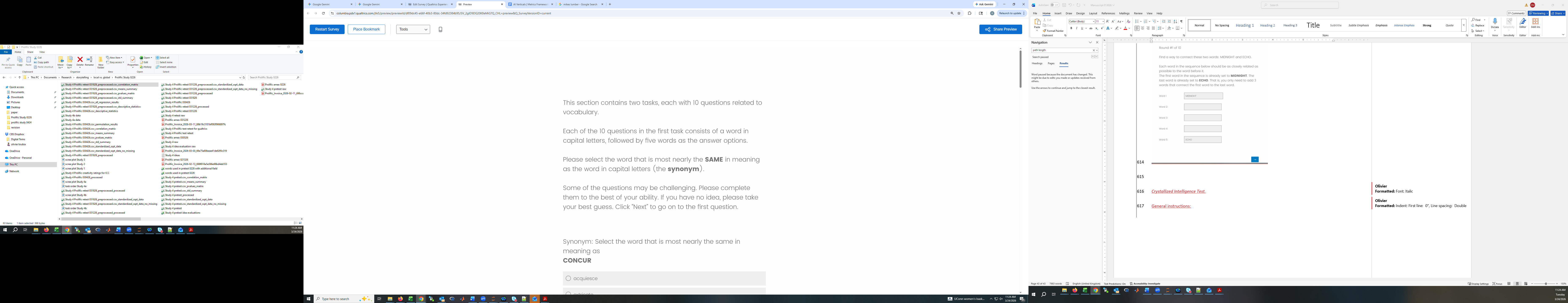


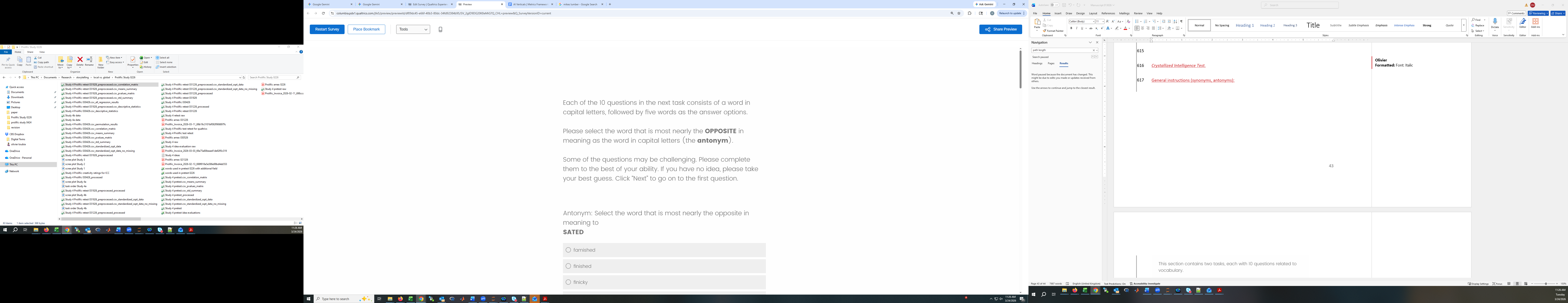


Task-level instructions (synonyms, antonyms):


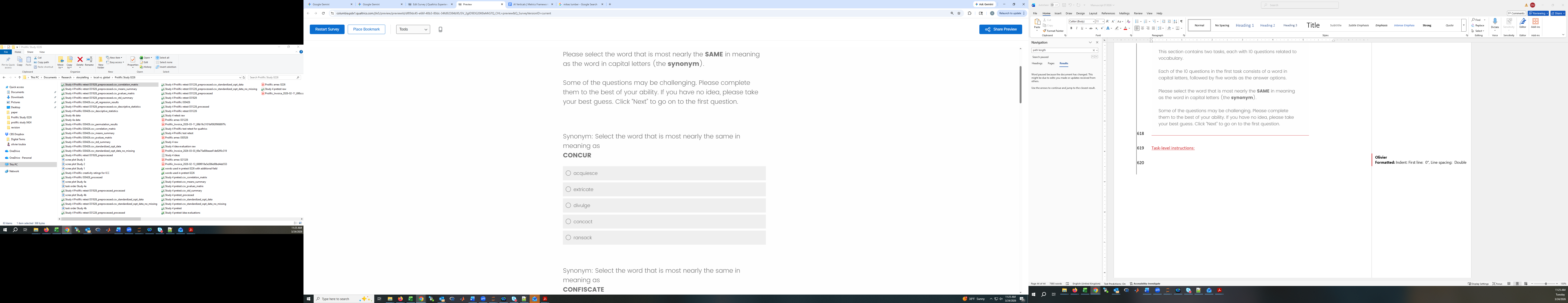


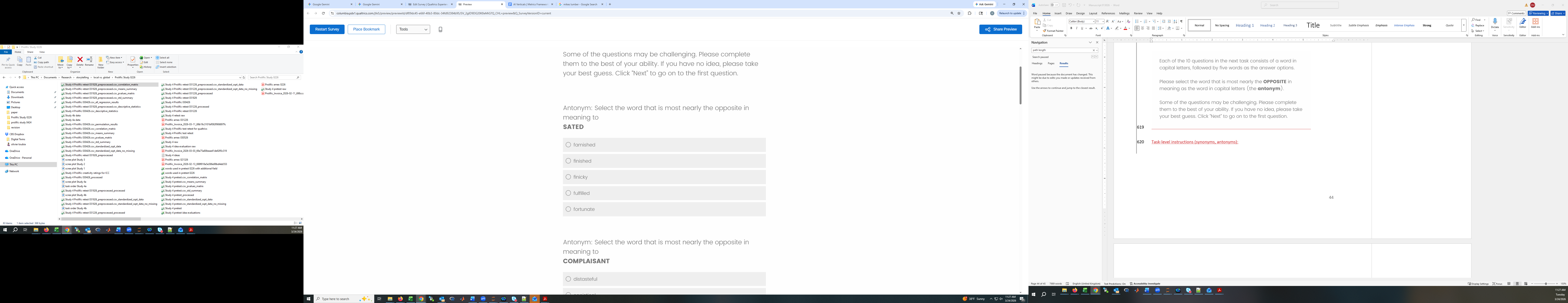


*Idea Generation.*

General instructions:


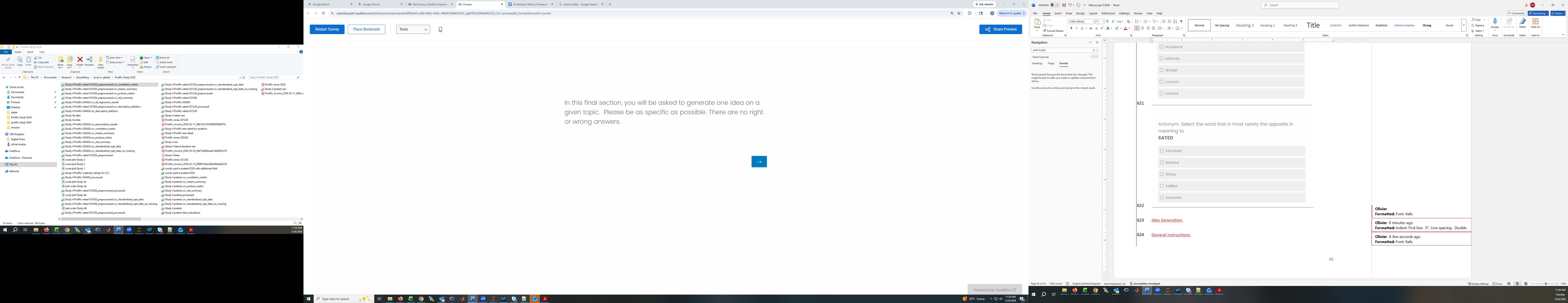


Task-level instructions:


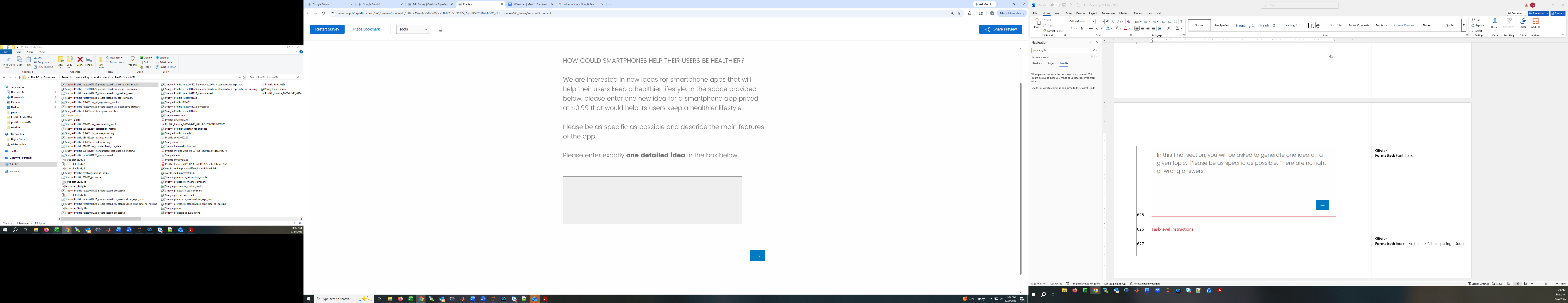


*Creativity Ratings for Unusual Uses Test.*

General instructions:


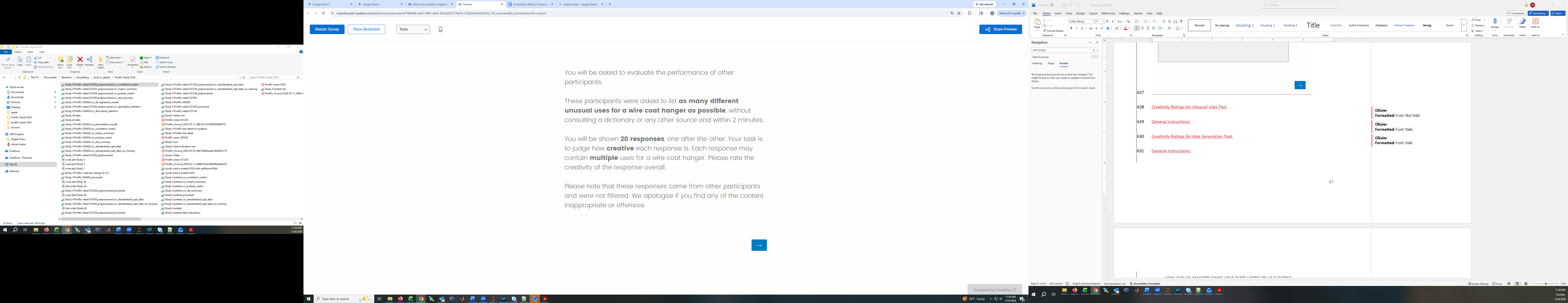


Task-level instructions:


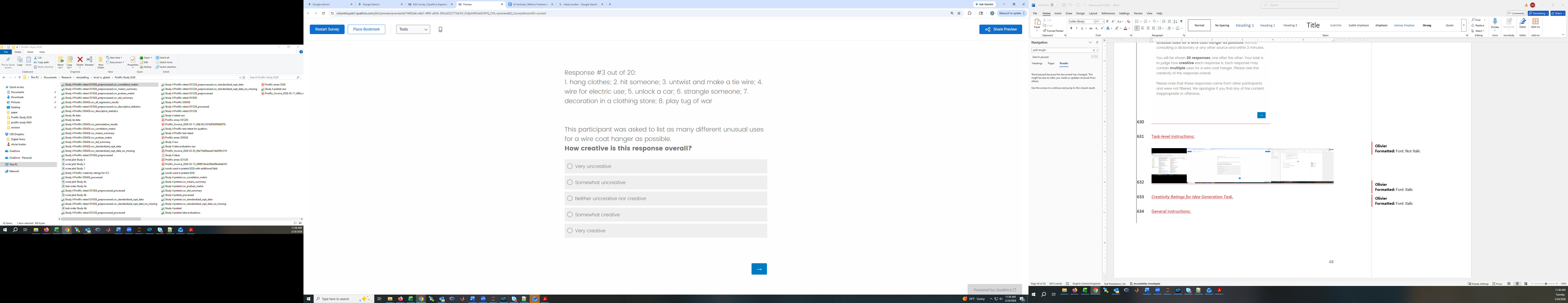


*Creativity Ratings for Idea Generation Task.*

General instructions:


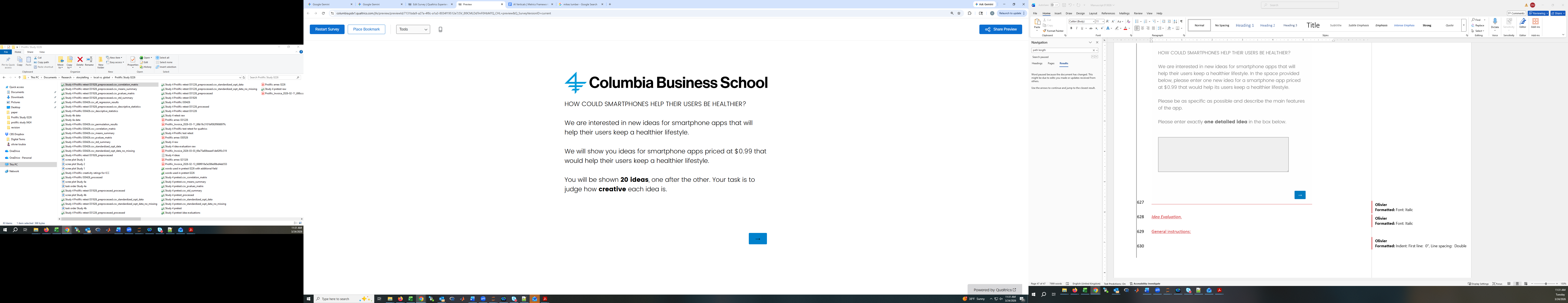


Task-level instructions:


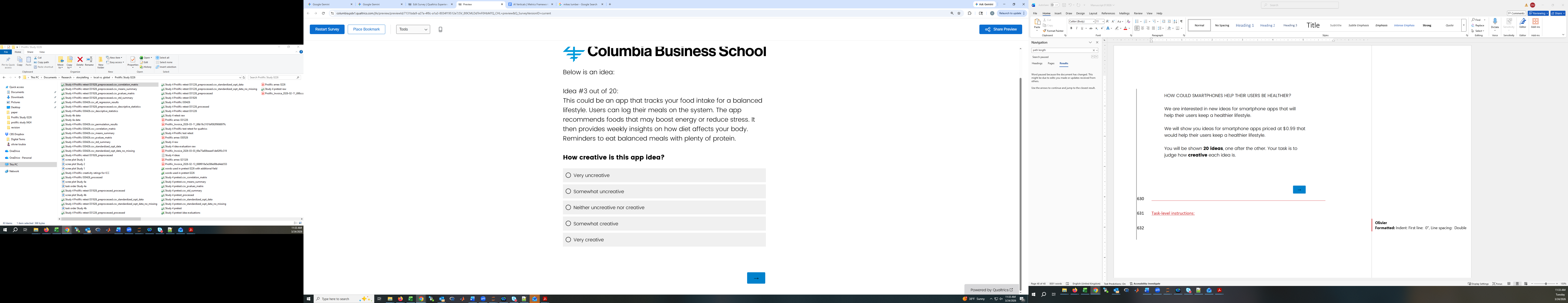

Supplement: S1 Text — (DOCX) [file pone.0352328.s001.docx]
